# Extracellular vesicles from cytokine-treated human pancreatic ductal cells enhance HLA class I expression in beta cells

**DOI:** 10.3389/fimmu.2026.1788321

**Published:** 2026-07-23

**Authors:** Neslihan Erdem, Heather N. Zook, Janine C. Quijano, Nathaniel P. Hansen, Joanna Palade, Eunjin Oh, Jacob Mares, Cecile Donohue, Jose A. Ortiz, Kevin Jou, Min Talley, Nagesha Guthalu Kondegowda, David Arribas-Layton, Rupangi C. Vasavada, Enrique Montero, Helena Reijonen, Debbie C. Thurmond, Patrick Pirrotte, Tijana Jovanovic-Talisman, Hsun Teresa Ku

**Affiliations:** 1 Department of Translational Research and Cellular Therapeutics, Arthur Riggs Diabetes and Metabolism Research Institute, City of Hope National Medical Center, Duarte, CA, United States; 2 Irell and Manella Graduate School of Biological Sciences, Beckman Research Institute, City of Hope National Medical Center, Duarte, CA, United States; 3 Integrated Mass Spectrometry Shared Resource, City of Hope National Medical Center, Duarte, CA, United States; 4 Early Detection and Prevention Division, Translational Genomics Research Institute, Phoenix, AZ, United States; 5 Department of Molecular and Cellular Endocrinology, Arthur Riggs Diabetes and Metabolism Research Institute, City of Hope National Medical Center, Duarte, CA, United States; 6 Department of Computational and Quantitative Medicine, City of Hope National Medical Center, Duarte, CA, United States; 7 Department of Immunology and Theranostics, Arthur Riggs Diabetes and Metabolism Research Institute, City of Hope National Medical Center, Duarte, CA, United States; 8 Department of Diabetes Immunology, Arthur Riggs Diabetes and Metabolism Research Institute, City of Hope National Medical Center, Duarte, CA, United States; 9 Department of Cancer Biology and Molecular Medicine, Beckman Research Institute, City of Hope National Medical Center, Duarte, CA, United States

**Keywords:** ductal cells, extracellular vesicles, HLA class I, human pancreas, proinflammatory cytokines

## Abstract

**Introduction/objective:**

Type 1 diabetes (T1D) is an autoimmune disease characterized by the loss of insulin-producing beta cells and has no cure. The role of cell-cell interactions in T1D disease progression remains poorly understood. Beta cells develop adjacent to ductal cells and both cell types undergo changes during T1D progression, suggesting a potential intercellular communication. Extracellular vesicles (EVs) are established mediators of intercellular communication, but whether human ductal cells secrete EVs that influence beta cells remains unknown. We hypothesize that exposure of human ductal cells to T1D-associated cytokines alters their EV cargo and that EVs produced by cytokine-exposed human ductal cells modulate gene and protein expression in beta cells.

**Methods:**

Human exocrine tissues from non-diabetic cadaveric donors were cultured in a 3D suspension to support ductal cell survival. Cells were treated with TNF-α, IL-1β, and IFN-γ or vehicle control for 48 hours and analyzed for proinflammatory-related gene expression and viability by qRT-PCR and Trypan blue exclusion, respectively. EVs from cytokine- and vehicle-treated ductal cells were isolated using size exclusion chromatography and characterized by dot blot, transmission electron microscopy, and nanoparticle tracking analysis. The effects of EVs on EndoC-βH1 human beta cells were assessed with qRT-PCR, RNA-sequencing, and flow cytometry; the effects of EVs on primary human beta cells were assessed with immunofluorescence microscopy. EV cargo was analyzed by proteomics and transcriptomics.

**Results:**

EVs from cytokine-treated ductal cells were internalized by EndoC-βH1 beta cells, and compared to control ductal EVs, increased HLA Class I gene and protein expression in EndoC-βH1 cells. In primary human beta cells, EVs from cytokine-treated ductal cells, compared to control ductal EVs, also increased expression of HLA Class I. EV cargo from cytokine-treated versus control ductal cells was enriched for transcripts associated with inflammatory and antigen-presentation-related pathways, including *CXCL9, CXCL10, CXCL11, IDO1*, and *GBP4*.

**Conclusion:**

Exposure of T1D-associated cytokines to primary human ductal cells altered cargo of secreted EVs. EVs from cytokine-treated ductal cells upregulated HLA Class I expression in EndoC-βH1 and primary human beta cells. Our results suggest a previously unknown ductal cell communication pathway with a potential implication for modulating beta cells during T1D progression.

## 1 Introduction

Type 1 diabetes (T1D) is an autoimmune disease characterized by the destruction of insulin-producing beta cells in the endocrine pancreas (1, 2). T1D is primarily driven by autoreactive immune cells and proinflammatory cytokines (3–6). However, beta cells also contribute to their own destruction in T1D by upregulating human leukocyte antigen (HLA) Class I molecules that are essential for recognition by cytotoxic T cells, thereby exacerbating immune-mediated damage (7–9). Recently, anti-CD3 antibodies that target immune cells have been shown to delay T1D onset (10–14); however, a cure remains elusive, indicating gaps in our understanding of the disease mechanisms.

Growing evidence suggests an association between exocrine pancreas, which comprises ductal and acinar cells, and T1D disease progression (15, 16). For example, human ductal cells treated with T1D-associated cytokines, IFN-γ and IL-1β, produce nitric oxide, which damage beta cells (17). Recent single-cell RNA sequencing analyses (18, 19) reveal that human ductal cells acquire a proinflammatory signature when exposed to IFN-γ, IL-1β, and TNF-α. Thus, cytokines in the T1D microenvironment may affect ductal cells, which in turn may influence beta cells. However, how ductal cells communicate with and modulate other cells remain poorly understood.

Extracellular vesicles (EVs) are key mediators of intercellular communication, carrying proteins, nucleic acids, and lipids that influence recipient cells (20, 21). EVs are known to regulate immune responses, inflammation, and cell death (22, 23). In T1D, EVs from endocrine islets contain autoantigens (24, 25); when islets are exposed to cytokines (TNF-α, IL-1β, and IFN-γ), EV cargo becomes enriched with proinflammatory (26) and TNF signaling molecules (27) that have been associated with immune cell recruitment and islet dysfunction (26). Past research on EVs from the exocrine pancreas has primarily focused on pancreatic ductal cell carcinoma and immortalized cell lines (28, 29). EVs secreted from primary human ductal cells have not been characterized and their impact on beta cells remains unknown.

In this study, we tested the hypotheses that 1) primary human ductal cells secrete EVs with distinct cargo after exposure to T1D-associated cytokines, and 2) EVs from cytokine-exposed ductal cells modulate EndoC-βH1 human beta cells and primary human islet beta cells. We characterized the EVs produced by ductal cells after 48 hrs with or without cytokines and found that 1) EVs from cytokine-treated ductal cells, compared to controls, upregulated HLA Class I molecules in human EndoC-βH1 beta cells and primary human islet beta cells; and 2) EV cargo from cytokine-treated compared to vehicle-treated ductal cells was enriched for mRNAs of inflammatory mediators. These findings demonstrate a previously unrecognized ductal cell-mediated communication with potential implication in T1D progression.

## 2 Materials and methods

### 2.1 Generation of EV-depleted KnockOut serum replacement

EV depletion of KnockOut Serum Replacement (Thermo Fisher Scientific, 10828-028) was carried out according to a published protocol (30). Briefly, KnockOut Serum Replacement (KSR) was ultracentrifuged at 120, 000 x g for 18 hrs at 4°C using SW 32 Ti rotor with Beckman Coulter Optima XPN Ultracentrifuge. The supernatant from each tube was recovered, aliquoted, and stored at -20°C.

### 2.2 Primary human pancreatic ductal cells

This study uses deidentified human cadaveric pancreatic tissues through the Southern California Islet Cell Resource Center (SC-ICRC) at the City of Hope (**Supplementary Table 1**). City of Hope Institutional Review Board did not require the study to be reviewed or approved by an ethics committee because deidentified cadaveric tissues do not meet the definition of human subjects research as set forth at 45 CFR 46.102(e). Islet-depleted pancreatic exocrine tissues were dissociated into single cell suspension and cryopreserved according to published protocols (31–33). Briefly, exocrine tissues were rinsed in cold PBS, resuspended in Dulbecco’s phosphate-buffered saline (PBS) (Corning, 21-031-CV) containing 0.1% bovine serum albumin (BSA) (Sigma-Aldrich, A8412), 2-4 mg/mL collagenase B (Sigma-Aldrich, 11088831001), and 2, 000 U/mL DNase I (Millipore Sigma, 260913), and incubated in a water bath set to 37 °C, with gentle disruption using a 16½ G syringe needle. Single-cell suspension was obtained by filtering through 100 μm and 40 μm nylon meshes. Dissociated single-cell suspensions were quantified, gently resuspended in Cryostor CS10 (Biolife Solutions, 210102), and frozen using a CryoMed™ Controlled-Rate Freezer (Thermo Scientific, Waltham, USA). Once frozen, the vials were transferred to a liquid nitrogen tank for long-term storage.

Cryovials were thawed and cells were cultured (32, 33). Briefly, frozen vials were placed in a 37 °C water bath for ~2 min and cells were gently transferred to a conical vial. Cells were washed once with warm PBS supplemented with 0.1% BSA, penicillin-streptomycin (PS), and 2, 000 U/mL DNase 1, then gently resuspended in suspension culture medium consisting of DMEM/F12, 10% EV-depleted KnockOut Serum Replacement (ED-KSR), PS, nicotinamide, Noggin, epidermal growth factor (EGF), A83-01, R-spondin1 (RSPO1), gastrin II sulfated, and Y-27632 (**Supplementary Table 2**). Cells were finally plated in ultra-low binding plates or flasks and incubated at 37 °C in a humidified 5% CO_2_ atmosphere.

For the initial overnight incubation (up to 16 hrs), Y-27632 was included in the culture medium. On culture day 1, cells were collected and washed with warm DMEM/F12 containing 0.1% BSA and PS. Live cells were enriched using Histopaque 1077 (Sigma-Aldrich, 10771-100ML) density gradient medium and counted using a hemocytometer and trypan blue exclusion. After live cell enrichment, cells were resuspended in freshly prepared, pre-warmed suspension medium without Y-27632 and were plated at 3 x 10^5^ cells per 1 mL media in ultra-low binding culture plates. Cells were incubated for up to 48 hrs at 37 °C in a humidified 5% CO_2_ atmosphere.

Graded doses of T1D-associated proinflammatory cytokines (TNF-α, IL-1β, and IFN-γ) (**Supplementary Table 3**) were prepared in suspension media and applied to day 1 ductal cells for 48 hrs.

In EV function experiments, “media only” refers to unconditioned culture medium composed of DMEM/F12, 10% ED-KSR and PS, incubated for 48 hrs at 37 °C in a humidified 5% CO2 atmosphere without any cells.

### 2.3 Primary human pancreatic islets

Islets from deidentified non-diabetic cadaveric donors were cultured in media containing RPMI-1640 supplemented with 5.5 mM glucose, 10% fetal bovine serum (FBS), and 1% PS overnight at 37 °C in a humidified 5% CO_2_ atmosphere (34, 35). After overnight incubation, human islets were trypsinized for 10 min with intermittent pipetting, and cells from 100 islet equivalents (IEQ) per well were plated on 8-well chamber slides initially in a small volume (50 µL) of EV-free media containing RPMI-1640 supplemented with 5.5 mM glucose, 10% EV-free FBS (Gibco, A2720803), and 1% PS for 2 hours to allow attachment. After 2 hours, EV-free media was added to complete the wells to 400 µL and cultured overnight at 37 °C in a humidified 5% CO_2_ atmosphere (34, 35). After overnight incubation, islets were treated with EVs as described in section 2.10.

### 2.4 EndoC-βH1 cells

The cell culture methods for EndoC-βH1 human beta cell line (Human Cell Design, Toulouse, France) were described previously (36, 37). Briefly, EndoC-βH1 cells were cultured onto 1% ECM-fibronectin (2 µg/mL) coated culture wells (Millipore Sigma, ECM, E1270; Fibronectin, F1141) in DMEM that contained 5.6 mM D-glucose (Thermo Fisher Scientific, 11885-084), 2% Fatty Acid Free heat shock BSA powder (Equitech, BAH66), 50 µM 2-mercaptoethanol, 10 mM nicotinamide (CalBiochem, 481907), 5.5 µg/mL transferrin (Millipore Sigma, T8158), 6.7 ng/mL sodium selenite (Millipore Sigma, S1382), and 100 U/mL penicillin, 100 μg/mL streptomycin (PS) (36, 37). All culture cells were incubated at 37 °C in a humidified 5% CO_2_ atmosphere.

### 2.5 Conventional and microfluidic quantitative (q)RT-PCR

Conventional qRT-PCR was performed as previously described (32). Briefly, suspension cultured cells were collected, washed, and lysed using RLT buffer supplemented with β-mercaptoethanol, and stored at -80 °C. Total RNA extraction was performed using the RNeasy Micro Kit (Qiagen, 74004) or Mini Kit (Qiagen, 74104) according to the manufacturer’s instructions. Complementary DNA (cDNA) was generated using QuantiTect Reverse Transcription Kit (Qiagen, 205313) or Omniscript Reverse Transcription Kit (Qiagen, 205113) according to the manufacturer’s protocol and was stored at -20 °C for up to 1 year. Commercially available predesigned primers and TaqMan Probes (Thermo Fisher Scientific, Waltham, USA) (**Supplementary Table 4**) were used to analyze gene expression. Two or three technical replicates were used in all PCR runs, in addition to negative controls (water). Each PCR reaction contained a total of 10 µL volume per well and was placed in a MicroAmp Optical 384-well reaction plate (Applied Biosystems, 4309849) covered with an adhesive film. Thermal cycling was performed on the Applied Biosystems ViiA7 (Thermo Fisher): 95 °C for 2 min, 45 cycles of 94 °C for 30 s, 60 °C for 60 s, 72 °C for 10 s, and finally 72 °C extension for 120 s followed by 4 °C. Results are expressed as the relative expression level, where *ACTB* was used as internal control for normalization.

Microfluidic qRT-PCR was performed as previously described (31, 32), with some modifications. Briefly, cDNA generated from EndoC-βH1 cells were collected in reaction buffer (CellsDirect One-Step qRT-PCR Kit, Invitrogen, 11753100) containing Taqman probes and pre-amplified (14 cycles) according to the manufacturer’s instructions (Fluidigm Biomark, now known as Standard Tools, San Francisco, USA). Pre-amplified cDNA was loaded onto a 48.48 Dynamic Array system using the NanoFlex integrated fluidic circuit (IFC) controller (Fluidigm Biomark IFC Controller MX, San Francisco, USA). Threshold cycle (Ct) was used to measure fluorescence intensity. Ct values were determined by the BioMark PCR analysis software (Fluidigm BioMark, San Francisco, USA) and expressed as relative expression (delta Ct) of the gene of interest to internal control gene (*ACTB*). Negative (water) and positive (cDNA from adult human pancreatic cells) controls were included in all experiments.

### 2.6 EV isolation

Ductal cells were seeded at the density of 3x10^5^ cells/mL at day 1 (D1) post-thawing in 3D suspension culture medium with ED-KSR as described above. At day 3 (D3), EVs were isolated from conditioned media (CM) as described previously (28, 38). Briefly, CM was collected and cleared from dead cells and debris by centrifugation at 300 x g for 10 min. CM was concentrated to 0.5 mL by loading into Vivaspin 20–100 kDa concentrators (Cytiva, 28-9323-63) equilibrated with 0.5 mg/mL IgG-free BSA in PBS and centrifugated at 1000 x g in 10 min increments. The concentrated supernatant (CS) was subsequently loaded onto a qEVoriginal 35 nm column (Izon, SP5) equilibrated with PBS for size exclusion chromatography (SEC), per manufacturer instructions. Fractions (F)1 to F9 were collected (~500 µL per fraction) and the absorbance at 280 nm wavelength was measured using NanoDrop Spectrophotometer in non-lysed SEC fractions to check for protein contamination in each fraction. F1-3 (EVs) and F7-9 (largely soluble protein fraction) were pooled for downstream analysis in designated experiments.

### 2.7 Dot blot

Eluted SEC fractions from qEVoriginal 35 nm column were blotted (2 μL per fraction) onto 0.2 μm nitrocellulose membranes (Thermo Fisher Scientific, 77012) and allowed to dry for 30 min. Membranes were blocked with Intercept Blocking Buffer (Li-Cor Biosciences, 927-60001) for 1 hr at room temperature and then incubated with primary antibodies overnight at 4 °C. Membranes were washed four times for 5 min with Tris buffered saline (TBS) supplemented with 0.2% Tween 20 (TBST) at room temperature before incubation with secondary antibodies for 1 hr at room temperature. All antibodies were prepared in Intercept Blocking Buffer supplemented with 0.2% Tween 20. Membranes were washed four times for 5 min with TBST prior to detecting fluorescence signals using the Li-Cor Odyssey CLx imaging system (Li-Cor Biosciences, Lincoln, USA). Antibodies used in this study are listed in **Supplementary Table 5**.

Sypro Ruby stain (Thermo Fisher Scientific, S11791) was used to detect the total protein amount, per manufacturer instructions. Sypro Ruby stained membranes were imaged on a Bio-Rad Chemidoc imaging system.

### 2.8 Nanoparticle tracking analysis

EV concentration and size distribution were determined by NTA using an NS300 Nanosight instrument (Malvern Panalytical; Malvern, UK). Samples were diluted to 1:10 and 1:25 in 1 mL PBS and injected into the sample chamber using an automated syringe pump, with the syringe flow rate set at 30 (arbitrary units). Automatic settings were applied for blur and minimum track length. For capture settings, screen gain was set to 5, camera level to 12, and temperature to 25 °C. For analysis settings, screen gain was set to 10, and the detection threshold was adjusted between 2 and 5 depending on the donor. Three 60-second videos were captured at 25 frames per second for each sample, and EV concentrations were averaged using the NanoSight NTA 2.3 software.

### 2.9 Transmission electron microscopy

EV samples (5 µL) were absorbed onto a 200-mesh grid that is glow-discharged and coated with carbon and Formvar (Ted Pella, 01801). Samples were prepared using conventional negative staining with 1% (w/v) uranyl acetate (Electron Microscopy Sciences, 22400-1). Images were captured using an FEI Tecnai 12 transmission electron microscope equipped with a Gatan OneView CMOS camera.

### 2.10 Treatment of EndoC-βH1 cells and primary human islets with EVs

To standardize the amount of EVs given to EndoC-βH1 cells and primary human islets, quantification of total EV protein content was performed on small aliquots of EV samples (21, 39–41). EV samples were lysed in RIPA buffer (Thermo Fisher Scientific, 89900) supplemented with 1x protease inhibitor (Sigma-Aldrich, 8820) and 1x phosphatase inhibitor (Roche, 04-906-845-001) and incubated on ice for 30 min. Protein concentration was determined using Micro bicinchoninic acid (MicroBCA) protein assay kit (Thermo Fisher Scientific, 23235). It was estimated that 50 µg/mL total EV protein content corresponded to ~7.2 x 10^8^ particles/mL (*N* = 3 donors used for NTA in characterization assays). Non-lysed intact EVs with doses equivalent to 1-50 µg/mL total EV protein content were applied to EndoC-βH1 cells or primary human islets, which were then incubated at 37 °C in a humidified 5% CO_2_ atmosphere for 48 hrs prior to downstream analysis.

### 2.11 Bulk RNA-seq sequencing of EndoC-βH1 cells

RNA quality control (QC) was performed using the Agilent Tapestation RNA Tape to confirm that RNA integrity number (RIN) was above 7. A total of 250 ng of RNA per sample was used for sequencing library preparation with the KAPA mRNA HyperPrep Kit (Roche, 08098123702), following the manufacturer’s protocol. Final libraries were validated using the Agilent Bioanalyzer DNA High Sensitivity Kit and quantified with Qubit. Sequencing was performed on the NovaSeq X Series platform using the 25B reagent kit (300 cycles). NovaSeq Control Software 1.2.2.48004 was used for sequencing, and Illumina BCL Convert v4.2.7 was employed to convert base call (BCL) files into FASTQ format.

RNA-seq reads were trimmed to remove sequencing adapters using Trimmomatic (42) and polyA tails using FASTP. The processed reads were mapped back to the human genome (hg38) using STAR software (v. 2.6.0.a) (43). The HTSeq software (v.0.11.1) (44) was applied to generate the count matrix, with default parameters.

Differential expression analysis was performed by normalizing raw read counts to expression values using the trimmed mean of M-values (TMM) normalization method implemented in edgeR. Specifically, for paired comparisons between experimental conditions, generalized linear models were employed to identify differentially expressed genes (DEGs), with TMM-normalized expression levels serving as the dependent variable and experimental condition as the independent variable. Pairing factors (e.g., individual) were included in the model to minimize confounding effects.

Gene set enrichment analysis (GSEA) was performed on a broader set of genes that passed *p* < 0.05 due to the finding that only three genes passed FDR cutoff in DEGs. GSEA algorithm was implemented using clusterProfiler (v.4.14.3) package in R (45–48), where a ranked list of whole genes according to their log_2_ fold change (FC) and *p*-values are provided.

### 2.12 Flow cytometry

For live/dead cell discrimination, EndoC-βH1 cells were washed with PBS and incubated with Zombie Violet Dead Cell Stain (Biolegend, 423113) diluted 1:1, 000 in PBS for 15 min at room temperature. Cells were washed with PBS and resuspended in flow cytometry buffer consisting of PBS with 2% FBS. For surface marker detection, cells were incubated with fluorophore-conjugated monoclonal antibodies (**Supplementary Table 5**) in 100 μL of flow cytometry buffer at 4 °C for 30 min in the dark. After staining, cells were washed twice, centrifuged at 300 x g for 5 min, and resuspended in 200 μL of flow cytometry buffer.

Flow cytometry was performed on a Attune Nxt Acoustic Focusing Flow Cytometer System (Thermo Fisher Scientific, Waltham, USA). Data were analyzed using FlowJo v.10 (BD Biosciences, Franklin Lakes, USA), and mean fluorescence intensities (MFI) were quantified in cells.

### 2.13 Immunofluorescence staining of primary human islets

Primary human islet cells were treated with EVs for 48 h, fixed in 4% paraformaldehyde (PFA) for 15 min and blocked for 1 hr at room temperature with blocking buffer (PBS supplemented with 10% donkey serum and 0.1% Tween 20). After blocking, cells were incubated overnight at 4 °C with primary antibodies diluted in blocking buffer (**Supplementary Table 5**), washed three times with PBS supplemented with 0.1% Tween 20, and incubated with secondary antibodies diluted in blocking buffer (**Supplementary Table 5**) for 1 h at RT, followed by 10 min incubation with DAPI to stain nuclei. Coverslips were mounted using Vectashield Plus Antifade Mounting Medium (Vector Laboratories, H-1900) for imaging.

Images of representative areas were acquired using Zeiss LSM 900 confocal microscope equipped with Plan-Apochromat 20x/0.8 or 40x/0.95 objectives. The pinhole was set to 1 Airy unit. Images were processed using Zeiss ZEN Blue software (Carl Zeiss, Germany).

To quantify signal intensity, all cells on chamber slides were imaged using a Zeiss Axioscan 7 slide scanner equipped with Plan-Apochromat 20x/0.8 objective. Images were analyzed using QuPath v4.2. Individual cells across the entire slide were identified using the cell detection tool based on DAPI+ nuclei. Beta cells were identified by C-peptide positivity using the object classification tool. Thresholds for marker positivity were established using negative control images stained with only secondary antibodies. The HLA-ABC mean fluorescence intensity (MFI) of each beta cell was exported from QuPath, and the values of these individual beta cells were then averaged to obtain a single representative HLA-ABC MFI per ductal EV donor.

### 2.14 Enzyme-linked immunosorbent assay

Quantification of TNF-α, IL-1β, IFN-γ was performed using commercially available ELISA kits: TNF-α (R&D Systems, HSTA00E), IL-1β (R&D Systems, HSLB00D), and IFN-γ (R&D Systems, HSDIF0). For each assay, standards and samples were diluted according to the manufacturer’s instructions. Antibodies precoated in 96-well plates were incubated with samples or standards, followed by sequential incubation with detection antibodies, enzyme conjugates, and substrate solutions. Absorbance was measured at 450 nm with wavelength correction at 570 nm using Tecan Spark Plate Reader (Tecan, Switzerland). Sample concentrations were calculated using the standard curve derived from the linear correlation equation generated by plotting the standards.

### 2.15 EV uptake assay

Isolation of EVs using Capto Core 400 resin was performed according to a published protocol (49). Spin columns (Bio-Rad, 7326008) and collection tubes were coated using PBS supplemented with 0.05% IgG-free BSA and 0.005% Tween-20 by rotating at room temperature for 10 min, followed by washing twice with 1 mL PBS. Capto Core 400 (Cytiva, 17372401) resin was diluted in PBS from 200 μL to 400 μL, transferred to a spin column and centrifuged at 500 x g for 3 min to remove the storage buffer. After two washes with 500 μL of PBS, 100 μL of concentrated supernatant was added to a capped spin column, and the slurry was incubated for 1 hr at room temperature on a rotator. Eluate (100 μL) was collected by centrifugation at 500 x g for 3 min. EV particle concentration was measured using NTA as described above.

Labeling of EVs with maleimide-CF568 was performed according to a published protocol (38). EVs were labeled in two parallel 150 μL reactions (total 300 μL of EVs). Briefly, 10 nmol of Maleimide-CF568 (Sigma, SCJ4600026) was added per 150 μL EV sample (24 x10^9^ particles/mL as measured by NTA), protected from light, and rotated for 1 hr at room temperature. Maleimide was quenched using 5 times molar excess L-glutathione (Sigma, 101179609) for 10 min. Excess maleimide-CF568 was removed through purification with a qEVsingle 35 nm column (Izon, ICS-35). Each 150 μL of labeled EVs was loaded onto qEVsingle 35 nm column, 850 μL of flow through was discarded, three 200 μL fractions containing EVs were collected and concentrated to 100 μL per reaction by loading into 100 kDa concentrators (Amicon, UFC210024) equilibrated with 0.5 mg/mL IgG-free BSA in PBS. The two reactions were combined (200 µL total) and 125 µL of labeled EVs was added to EndoC-βH1 cells previously seeded onto 8-well chamber slides (200, 000 cells per well, total volume 250 µL per well) (Corning, 354118).

After 24 hr of incubation at 37 °C, cells were washed with PBS and fixed with 4%PFA for 15 min at room temperature. After two 10-min PBS washes, cells were blocked for 1 hr at room temperature with blocking buffer (PBS containing 10% donkey serum and 0.1% Tween 20), followed by overnight incubation at 4 °C with primary antibodies diluted in blocking buffer. Cells were washed three times with PBS containing 0.1% Tween 20 and incubated with secondary antibodies for 1 hr at room temperature, followed by a 10-min incubation with DAPI (Invitrogen, D3571) to stain nuclei. Coverslips were mounted using Vectashield Plus Antifade Mounting Medium (Vector Laboratories, H-1900) for imaging. Antibodies used are listed in **Supplementary Table 5**.

Images were acquired using a Zeiss LSM 880 or Zeiss LSM 900 confocal microscope equipped with either a Plan-Apochromat 40x/1.4 oil immersion or Plan-Apochromat 63x/1.4 oil immersion objective. For Z-stack imaging, optical sections were captured at 0.2 µm intervals with the pinhole set to 1 Airy unit. Images were processed using Zeiss ZEN Blue or Black software (Carl Zeiss, Germany).

### 2.16 EV protein cargo analysis

EVs were lysed using a tip sonicator in 2% deoxycholate lysis buffer containing 50 mM ammonium bicarbonate and 1x protease and phosphatase inhibitor cocktail (Thermo Fisher Scientific, 78440). Lysates were clarified by centrifugation at 16, 000 x g and protein concentration was quantitated using the Pierce BCA Protein Assay Kit (Thermo Fisher Scientific, 23225). Samples were reduced by DTT (5 mM final concentration) for 45 min at room temperature and alkylated in the dark using iodoacetamide (20 mM final concentration) for 30 min at room temperature. Samples were diluted 2-fold and digested overnight with trypsin (Trypsin Gold, Promega, V5280) at an enzyme to substrate ratio of 1:50. Digests were quenched with trifluoric acid and then desalted using Waters Sep-Pak C18 columns. The resulting peptides were vacuum-centrifuged to dryness (50).

Mass spectrometry (MS) data were acquired using a U3000 RSLCnano liquid chromatography system (Thermo Fisher Scientific, Waltham, USA) coupled to an Orbitrap Eclipse mass spectrometer (Thermo Fisher Scientific, Waltham, USA) equipped with a FAIMS Pro device. Peptides (500 ng per sample) were directly loaded onto a 50 cm C18 EASY-Spray column (Thermo Fisher Scientific, ES903) and separated using a 2 hr reverse-phase chromatography gradient at a flow rate of 300 nl/min. MS1 scans were acquired in the Orbitrap at a resolution of 120K, followed by MS2 scans in the ion trap using higher-energy collisional dissociation (HCD) fragmentation with a normalized collision energy of 32%. Data acquisition was performed in a data-dependent mode, selecting the most abundant precursors from MS1 for fragmentation. Each scan cycle incorporated an alternating FAIMS compensation voltage (CV) of -40, -60, and -80, applied sequentially for 1 s each.

Raw spectra were searched against a human protein database (Swissprot, Human 2020) using Mascot (v2.6) search engine through Proteome Discoverer (v2.4). Protein digestion was set to trypsin and allowed for a maximum of 2 missed cleavages. For feature linking and mapping, retention time tolerance was set to 0 min and mass tolerance was set to 0 ppm, allowing for match between runs. Both the PSMs and peptides were filtered for a false discovery rate (FDR) <1% calculated via Percolator. Methionine oxidation was set as a variable modification and cysteine carbamidomethyl was set as a fixed modification. Protein abundances were normalized using variance stabilizing normalization in R (v4.4.0). A Student’s *t*-test was run on the normalized data to determine differentially abundant proteins.

### 2.17 EV RNA cargo analysis

RNAs were isolated using the Plasma/Serum Circulating and Exosomal RNA Purification Mini Kit (Norgen Biotek, 51000) according to the manufacturer’s instructions with slight modifications. Briefly, EVs were lysed at 60 °C with silica slurry in a 2-mercaptoethanol lysis buffer. Nucleic acids were precipitated with ethanol and RNA was captured onto the column, treated with DNase I (Norgen Biotek, 25710), washed, and eluted with RNase-free DNase-free ultra-pure water. RNA concentrations were determined using Quant-iT RiboGreen (Thermo Fisher, R11490), with the low-range standard curve and an Agilent Cytation plate reader.

Small RNA libraries were generated from EV RNAs using NEXTFLEX Small RNA Sequencing Kit V4 (Revvity, NOVA-5132-41), without the YRNA/tRNA blockers. Following ligation with 0.25x diluted 3’ and 5’ adenylated adapter, libraries were reverse transcribed, bead cleaned and amplified with 22 cycles of PCR. After a final round of bead-based size selection, library size and concentration were assessed with the D1000 High Sensitivity DNA kit on the Agilent’s TapeStation 4200 instrument. Whole transcriptome libraries were generated using SMARTer Stranded Total RNA-Seq Kit v3 - Pico Input Mammalian RNA (Takara Bio, 634485) and SMARTer RNA Unique Dual Index Kits (Takara Bio, 634452). First, RNAs were treated with HL-dsDNase (ArcticZymes, 70800-21) at 37 °C for 10 min, to remove residual DNA. RNA fragmentation was done at 94 °C for 2 min, followed by reverse transcription, and PCR with 5 cycles. Size selection and sample clean-up were done with AMPure XP magnetic beads (Beckman Coulter, A63882), and post ribodepletion libraries were amplified by PCR with 16 cycles. Library size was assessed with the D1000 High Sensitivity DNA kit and concentration was analyzed via qPCR with KAPA Complete Kit Universal (Roche, KK4824). Short read sequencing was carried out using Illumina’s NovaSeq X platform. Small RNA libraries were sequenced on v1 10B flowcells, with 1% PhiX spike in, at 170 pM final clustering concentration. Whole transcriptome RNA library pools were sequenced on NovaSeq X on v1 10B flowcells, with 1% PhiX spike in, at 150 pM final clustering concentration.

For small RNA-sequencing data analysis, the exceRpt Small RNA-seq pipeline was employed, which is a comprehensive and automated workflow designed for processing and analyzing small RNA-sequencing data (51). The exceRpt performs quality control, adapter trimming, alignment to multiple reference databases and quantification of small RNA species. The pipeline also filters and annotates reads based on their alignment characteristics, providing a detailed breakdown of small RNA biotypes. Default parameters were used, and the analysis was executed using exceRpt within a Singularity container to ensure reproducibility.

Whole transcriptome data were processed using an in-house pipeline at TGen. Unique molecular identifiers were pulled out to eliminate amplification bias, and adapter sequences were trimmed using cutadapt v4.4. Fastq-screen v0.15.3 was utilized to identify potential contamination, comparing sequences against a panel of genomes. Reads were aligned to the GRCh38 reference genome using STAR v2.7.10b, and the number of reads mapping to each gene was quantified with FeatureCounts. For downstream analysis, duplicate reads were removed, and genes with fewer than 10 counts in at least half the samples were filtered out. For normalization and differential expression analysis, the data were processed in R v4.4.0 using DESeq2. For Ingenuity Pathway Analysis (IPA) analysis, transcripts were filtered by *p*-value <0.05, and log_2_FC values were used to calculate pathway z-scores.

### 2.18 Statistical analysis

Statistical analyses and plots generation were performed using GraphPad Prism version 9.5 or 10. Results are presented as mean ± SEM or mean ± SD, as indicated in the figure legends. Unless otherwise specified, significance between two groups was determined using t-test, and significance among three or more groups was determined using one-way ANOVA. Statistical significance was defined as *p* < 0.05 and was indicated as follows: *p < 0.05, **p < 0.01, ***p < 0.001, ****p < 0.0001, and not significant (ns) *p* > 0.05. Additional details regarding statistical tests are provided in the figure legends.

## 3 Results

### 3.1 Human ductal cells respond to T1D cytokines in a dose-dependent manner

To study primary human ductal cells, we previously established a 3D suspension culture system using serum-free medium with defined growth factors (**Supplementary Table 2**) that support the survival of ductal cells (32). Human exocrine tissues from de-identified donors without apparent diseases were obtained from Southern California Islet Cell Resource Center at City of Hope, with an average age of 36 ± 10 years, body mass index of 25 ± 5 kg/m^2^, and hemoglobin A1c of 5.1 ± 0.3% (**Supplementary Table 1**).

**Figure 1:**
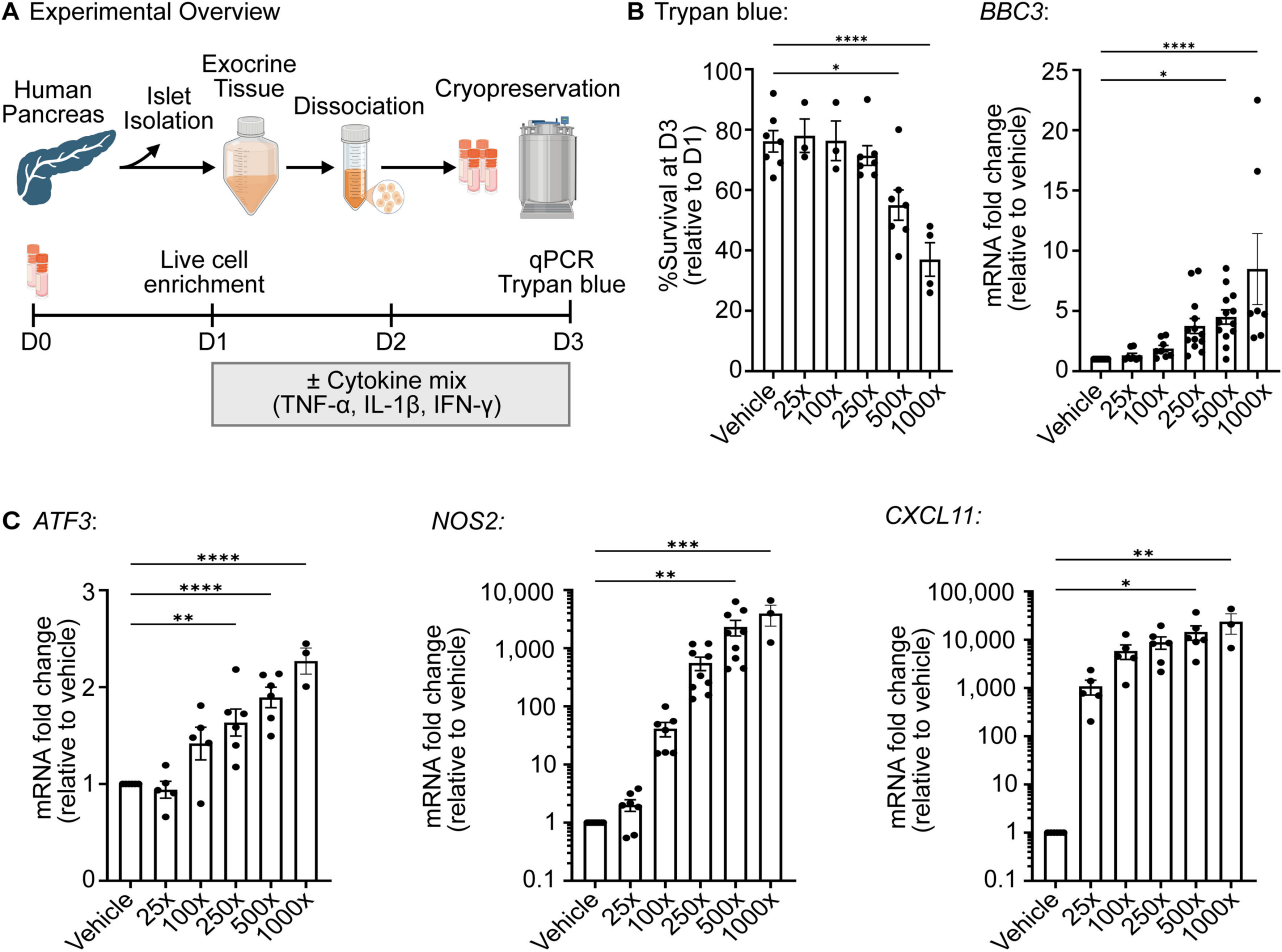
Primary human ductal cells from non-diabetic donors respond dose-dependently to proinflammatory cytokines *in vitro*. **(A)** Experimental workflow. Human ductal cells in a 3D suspension culture system were treated for 48 hrs with graded doses of a combination of proinflammatory cytokines. A dose of “250x” is defined as 250 IU/mL TNF-α, 25 IU/mL IL-1β, and 250 IU/mL IFN-γ; also see **Supplementary Table 3**. Created in BioRender. Erdem, N. (2026) https://BioRender.com/8dzogab. **(B)** Cytotoxicity effects on ductal cells were achieved by the doses of 500x and 1000x. (left panel) Quantification of live cells with trypan blue exclusion assay. Data represent the percentage of live cells after 48 hrs cytokine treatment (Day 3 compared to Day 1). *N* = 3–7 biological replicates from 3–6 unique donors. Each dot represents a donor, with the mean value derived from *n* = 4–5 technical replicates. (right panel) Conventional qRT-PCR for apoptosis marker *BBC3*. *N* = 7–13 biological replicates from 7–10 unique donors. Each dot represents a donor, with the mean value derived from *n* = 3 technical replicates. Data represent mean ± SEM. One-way ANOVA with Tukey’s test was used to determine significance. *p < 0.05, **p < 0.01, ***p < 0.001, and ****p < 0.0001. **(C)** Conventional qRT-PCR analysis for inflammatory response markers *ATF3*, *NOS2*, and *CXCL11*. *N* = 3–9 biological replicates from 3–7 unique donors. Each dot represents a donor, with the mean value derived from *n* = 3 technical replicates. Expression levels were normalized to housekeeping gene β-actin (*ACTB*) and are presented as fold change relative to vehicle control. Data represent mean ± SEM. One-way ANOVA with Tukey’s test was used to determine significance. *p < 0.05, **p < 0.01, ***p < 0.001, and ****p < 0.0001.

Exocrine tissues were dissociated into single-cell suspension, cryopreserved, thawed, and recovered overnight. On day 1, live ductal cells were enriched using density gradient centrifugation (32) and treated for 48 hrs with cytokines (**Supplementary Table 3**; **Figure 1A**). TNF-α, IL-1β, and IFN-γ, key mediators of immune responses in T1D (6, 52, 53), were used in this study. A combination of 1, 000 IU/mL TNF-α, 100 IU/mL IL-1β, and 1, 000 IU/mL IFN-γ (“1000x”) has been shown to exert killing effects on isolated human islet cells (54). Due to limited knowledge of cytokine effects on ductal cells, we first conducted dose-response experiments using 1000x as the highest dose and serial dilutions (**Supplementary Table 3**).

Trypan blue exclusion assay revealed that the 500x and 1000x cytokine doses significantly reduced live cell numbers compared to vehicle control (**Figure 1B**, left panel), indicating cytotoxicity. Quantitative (q) RT-PCR analysis confirmed this effect as evidenced by an upregulation of the apoptosis marker *BBC3* (55) in ductal cells treated with the 500x and 1000x cytokine doses (**Figure 1B**, right panel).

The inflammatory stress response genes *ATF3, NOS2*, and *CXCL11*, known downstream targets of cytokine signaling (56, 57), were upregulated by the 500x and 1000x doses compared to vehicle control (**Figure 1C**). However, 250x dose was sufficient to induce *ATF3* expression (**Figure 1C**), suggesting that the 250x dose elicits a cytokine-associated stress response without detectable cytotoxicity in ductal cells (**Figure 1B**).

EVs released from apoptotic cells differ in cargo content and function from those of healthy cells (27, 58–60). To better mimic non-apoptotic conditions, we selected the 250x dose (250 IU/ml TNF-α, 25 IU/ml IL-1β, and 250 IU/ml IFN-γ; 48h) for subsequent experiments. Human ductal cells treated with this 250x cytokine dose are referred to as “cyto-ductal cells”, and vehicle-treated cells as “ctrl-ductal cells.”

### 3.2 Characterization of extracellular vesicles isolated from conditioned media of ctrl- and cyto-ductal cells with size exclusion chromatography

**Figure 2:**
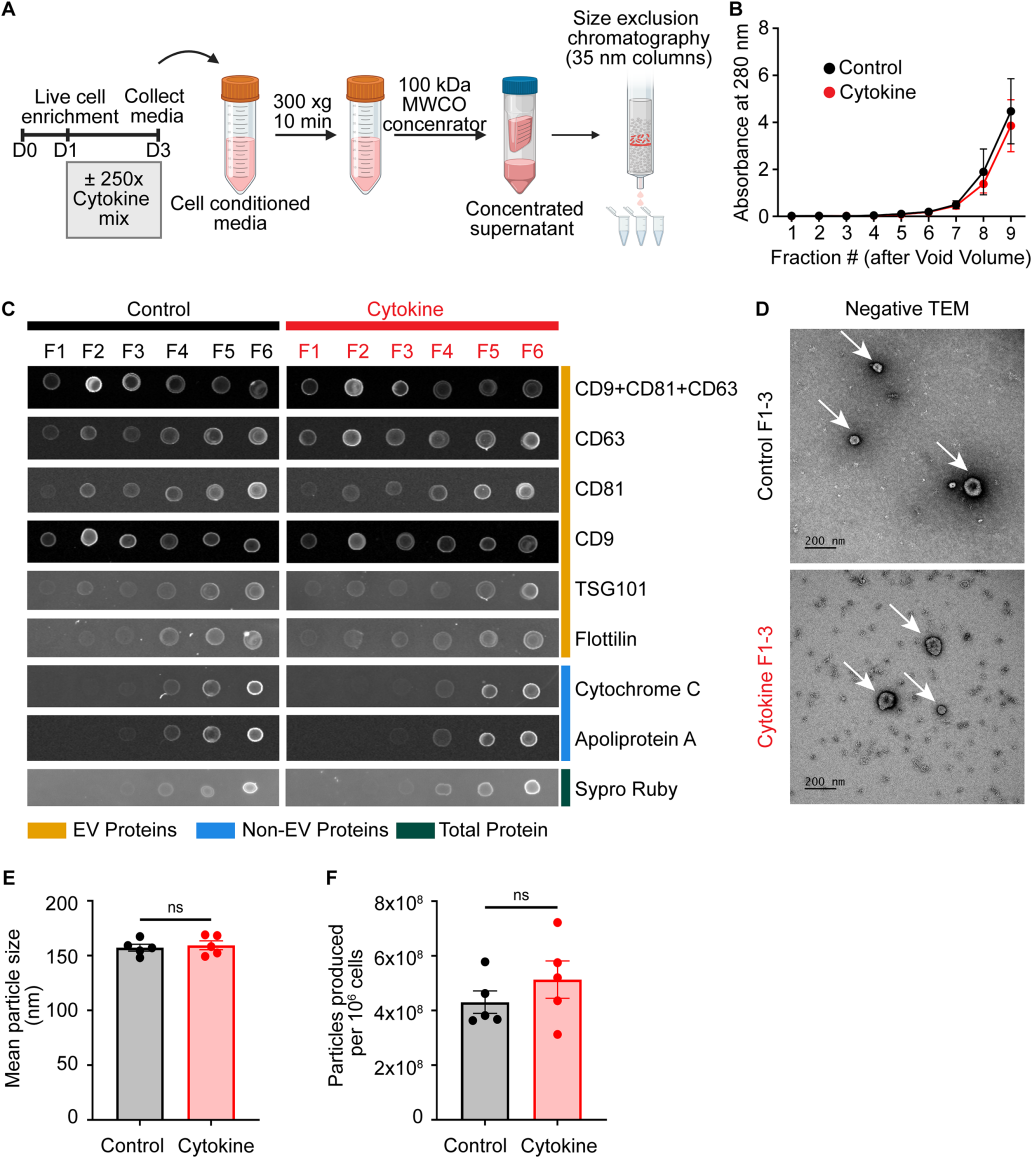
Extracellular vesicles (EVs) are detected in the conditioned media of primary human ductal cells treated with or without 250x cytokines. **(A)** Experimental workflow. EVs were isolated and purified using size exclusion chromatography (SEC) column. Created in BioRender. Erdem, N. (2026) https://BioRender.com/eciuqcm. **(B)** Detection of proteins in fractions eluted from SEC at the 280 nm absorbance wavelength using NanoDrop Spectrophotometer. *N* = 6 donors. Data represent mean ± SD. **(C)** EVs are purified in fractions (F)1, F2, and F3. Representative dot blots on F1 to F6 from a donor. EV markers used are CD81, CD63, CD9, Flottilin, and TSG101. Non-EV markers are Apolipoprotein A and Cytochrome **(C)** Total proteins were stained by Sypro Ruby. **(D)** Representative TEM image of EVs in combined F1, F2, and F3 (F1-3). Arrows indicate individual EV detected. The image was taken at a 30, 000x magnification. Scale bar = 200 nm. **(E)** Mean particle sizes from Nanoparticle Tracking Analysis (NTA). *N* = 5 biological replicates from 5 unique donors. Data represent mean ± SEM. Paired *t*-test was used to determine significance. ns: not significant, *p*>0.05. **(F)** Particle numbers from NTA were normalized per one million ductal cells plated. Data represent mean ± SEM from 5 unique donors. Paired t-test as used to determine significance. ns, not significant, *p*>0.05.

To characterize EVs from ctrl- and cyto-ductal cells, conditioned media were collected after 48 hrs of treatment and concentrated. Size exclusion chromatography (SEC) was used to isolate EVs from soluble proteins (**Figure 2A**). NanoDrop Spectrophotometer analysis at 280 nm showed that the majority of proteins eluted after fraction (F) 6 (**Figure 2B**).

EVs are expected to be enriched in the early fractions of SEC columns designed for EV isolation (28, 37, 38, 61, 62). We therefore characterized F1 through F6 using dot blot analysis. All conventional EV markers, CD9, CD81, CD63, TSG101, and Flotillin, were detected in F1 through F6 from the conditioned media of both ctrl- and cyto-ductal cells, although signals for TSG101 and Flotillin were weaker in F1 (**Figure 2C**). Importantly, Cytochrome C, a cytoplasmic marker, and Apolipoprotein A that can co-isolate with EVs, were detected in F4 to F6 (**Figure 2C**). Consistently, total protein staining using Sypro Ruby dye was more pronounced in F4 to F6 (**Figure 2C**). Taken together, these results demonstrate that fractions F1 to F3 are highly enriched for EV markers and largely free of non-specific proteins. We therefore pooled F1 to F3 (hereafter referred to as F1-3) for subsequent EV characterization.

Transmission electron microscopy (TEM) confirmed EV morphology, size, and membrane structure (21, 62) (**Figure 2D**). Nanoparticle tracking analysis (NTA) of F1–3 EVs from ctrl- and cyto-ductal cells (five donors) showed similar size distributions, with particles around ~120 nm being the most prevalent (**Supplementary Figure 1**; compare the black and red lines within each donor). Mean particle size (~150 nm) also did not differ between the two groups (**Figure 2E**). Normalized to the number of initially plated ductal cells, ~4.7 x 10^8^ particles were secreted per one million ductal cells over a 48-hr period (n=8 samples, 4 per group), with no difference between ctrl- and cyto-ductal EVs (**Figure 2F**). Taken together, these findings demonstrate that 250x cytokine treatment does not alter EV physical characteristics or quantity.

### 3.3 Purified Cyto-ductal (F1-3) EVs, at the dose of 50 µg/mL, increase expression of HLA Class I related genes in EndoC-βH1 beta cells

**Figure 3:**
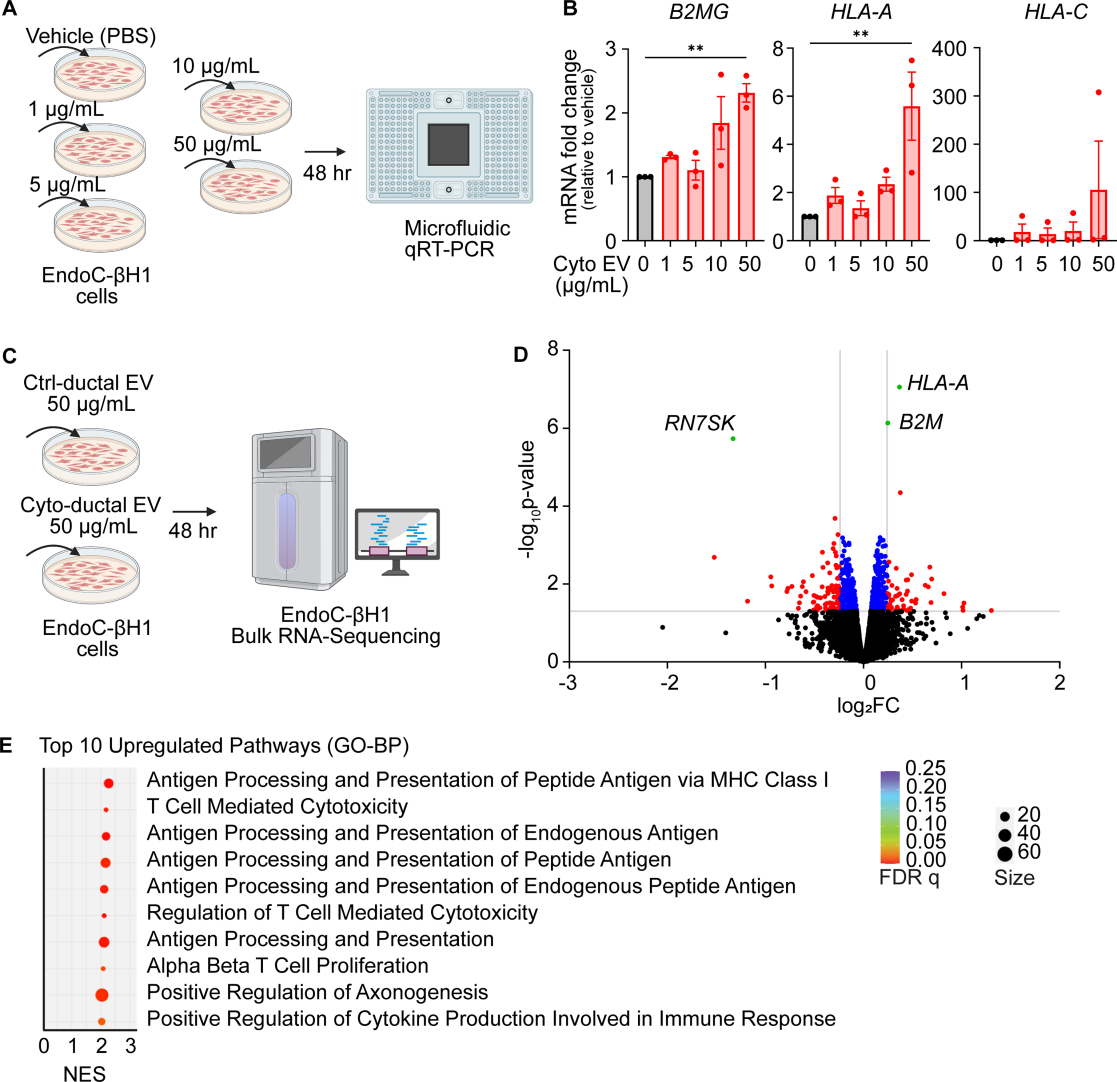
Human EndoC-βH1 beta cells, when treated with EVs from ductal cells exposed to 250x cytokines, upregulate genes involved in HLA Class I and antigen processing and presentation. **(A)** Experimental workflow. EndoC-βH1 cells were treated for 48 hrs with graded doses of cyto-ductal EVs purified from SEC (F1-3). Gene expression was analyzed by microfluidic qRT-PCR. Created in BioRender. Erdem, N. (2026) https://BioRender.com/yxdl9n8. **(B)** HLA Class I genes are upregulated by cyto-ductal EVs. *N* = 3 donors. Each dot represents a donor, with the mean value derived from n=3 technical replicates. Levels of mRNA expression were normalized to housekeeping gene β-actin (*ACTB*) and are presented as fold change relative to vehicle control. Data represent mean ± SEM. One-way ANOVA with Tukey’s test was used to determine significance. **p < 0.01. **(C)** Experimental workflow. EndoC-βH1 cells were treated with ctrl- and cyto-ductal EVs for 48 hrs, followed by bulk RNA-sequencing. Created in BioRender. Erdem, N. (2026) https://BioRender.com/y84cx4e. **(D)** Volcano plot showing differentially expressed genes (DEGs) in EndoC-βH1 cells treated with cyto- versus ctrl-ductal EVs. *N* = 4 donors. Each dot represents a single gene, with the log_2_ fold change (log_2_ FC) plotted against the -log_10_ (p-value). Genes with False discovery Rate (FDR) < 0.05, *p* < 0.05, |log_2_ FC| > 0.24 are shown in green. Genes with *p* < 0.05, |log_2_ FC| > 0.24 are shown in red. Non-significant genes are represented in black. The horizontal dashed line indicates the statistical significance threshold, and the vertical dashed lines represent the log_2_ FC cutoff. **(E)** Gene set enrichment analysis (GSEA) using Gene Ontology-Biological Processes (GO-BP) molecular signature database. NES indicates the normalized expression score, FDR indicates false-discovery rate.

To assess the biological effects of cyto-ductal EVs, we first utilized EndoC-βH1 beta cells, an immortalized line derived from a human embryo that retains many beta cell characteristics while providing a homogeneous genetic background (36). EndoC-βH1 cells were treated for 48 hrs with graded doses of cyto-ductal F1–3 EVs (1-50 µg/mL), and gene expression was examined (**Figure 3A**). EV dosing was normalized to total protein content of EVs, consistent with prior EV functional studies (21, 39, 41).

We examined genes involved in apoptosis, cell cycle regulation (**Supplementary Figure 2A**), inflammatory and anti-inflammatory responses (**Supplementary Figures 2B, C**), metabolism (**Supplementary Figure 2D**), and hypoxia (**Supplementary Figure 2E**) using microfluidic qRT-PCR; none showed significant changes after cyto-ductal EV treatment compared to PBS control. In contrast, HLA Class I genes *B2M* and *HLA-A*, but not *HLA-C*, were upregulated at 50 µg/mL EVs compared to PBS control (**Figure 3B**). However, *HLA-B* and HLA Class II genes (*HLA-DRA*, *HLA-DQA*, and *HLA-DQB*) were below detection limit (data not shown).

To compare the global gene expression effects of cyto- versus ctrl-ductal F1–3 EVs on beta cells, we performed bulk RNA-sequencing (bulk RNA-seq) on EndoC-βH1 cells treated with 50 µg/mL EVs from four donors for 48 hrs (**Figure 3C**). Of 735 genes meeting the significance threshold of *p ≤*0.05, only three genes passed the False Discovery Rate (FDR) cutoff (≤0.05; **Supplementary Dataset 1**), indicating largely similar transcriptome induced by cyto- versus ctrl-ductal F1–3 EVs. *HLA-A* (log_2_ fold change [log_2_ FC] = 0.367) and *B2M* (log_2_ FC = 0.247), previously identified in microfluidic qRT-PCR analysis (**Figure 3B**), were among the three genes (**Figure 3D**).

GSEA using the Gene Ontology Biological Process (GO-BP) molecular signature database identified “Antigen processing and presentation” and “T cell-mediated cytotoxicity” as top ranked pathways based on enrichment score in EndoC-βH1 cells exposed to cyto-ductal EVs versus ctrl-ductal EVs (**Figure 3E**; **Supplementary Dataset 2**). Overall, these results demonstrate that cyto-ductal EVs, compared to ctrl-ductal EVs, modestly upregulate HLA Class I genes in EndoC-βH1 cells.

### 3.4 Protein expression of HLA-ABC is increased in EndoC-βH1 cells and primary human beta cells treated with cyto-ductal EVs

**Figure 4:**
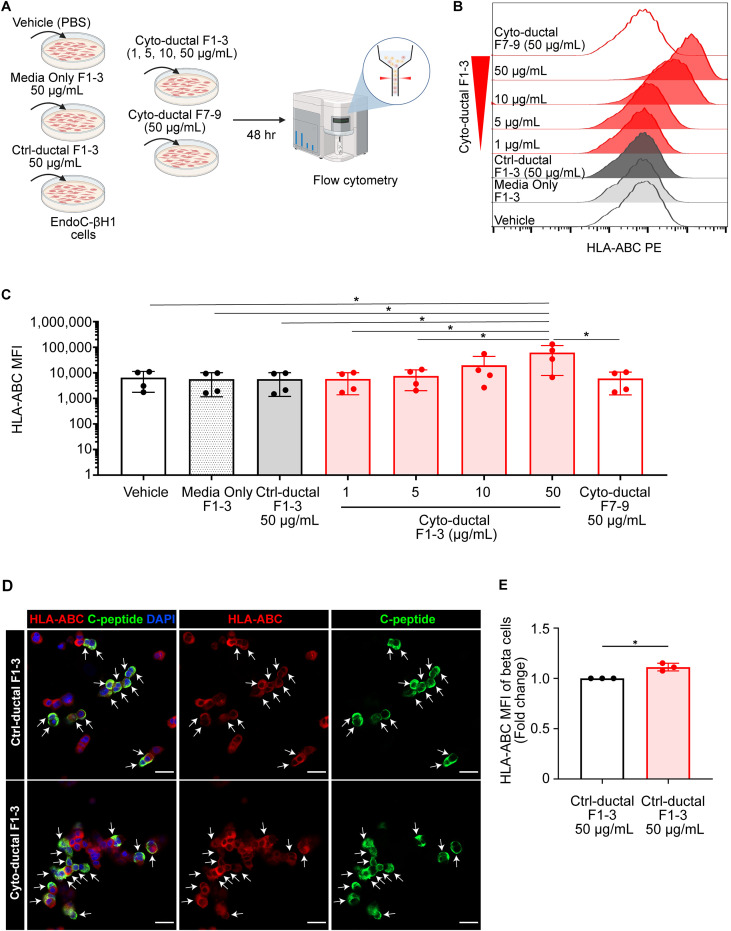
Cyto-ductal EVs increase surface protein expression of HLA-ABC on human beta cells. **(A)** Experimental workflow. EndoC-βH1 cells were treated for 48 hrs with vehicle, SEC fractions F1–3 from unconditioned culture media (“media only”), F1–3 ctrl-ductal EVs, F1–3 cyto-ductal EVs, or F7–9 proteins from conditioned medium of cyto-ductal cells. Flow cytometry was used to evaluate the surface expression levels of HLA-ABC. Created in BioRender. Erdem, N. (2026) https://BioRender.com/8k2vygs. **(B)** Representative histograms displaying HLA-ABC fluorescence intensity in each treatment group. **(C)** Quantification of mean fluorescence intensity (MFI) of HLA-ABC on EndoC-βH1 cells in each treatment group. *N* = 4 ductal EV donors. Each dot represents a ductal EV donor. Data represent mean ± SD. One-way ANOVA with Tukey’s test was used to determine significance. **p < 0.01. **(D)** Representative confocal microscopy images of primary human islet cells treated with ctrl- and cyto-ductal EVs for 48h hours. Cells were fixed, and stained with HLA-ABC, C-peptide, nuclei (DAPI) shown as red, green, and blue, respectively. White arrows highlight beta cells that are positive with C-peptide. Scale bar: 20 µm. **(E)** Quantification of HLA-ABC MFI of primary human islet beta cells (from two donors). Each dot represents *N* = 3 ductal EV donors. Data represent mean ± SD. Paired *t*-test was used to determine significance. *p < 0.05.

We assessed HLA-ABC expression on EndoC-βH1 cells by flow cytometry (gating strategy in **Supplementary Figure 3**). Cyto-ductal EVs at the 50 µg/mL dose, but not lower doses, significantly increased HLA-ABC mean fluorescence intensity (MFI) (**Figures 4A–C**). Controls included 1) vehicle PBS, 2) F1–3 isolated from unconditioned ductal cell media (“media only”), 3) F1–3 isolated from ctrl-ductal cells (“ctrl-ductal EVs)”, and 4) F7–9 isolated from cyto-ductal cells that largely contained soluble protein components; MFI of HLA-ABC showed no changes among these controls (**Figures 4B, C**). Next, the effect of cyto-ductal EVs (N=3 donors) on expression of HLA-ABC in primary human islet beta cells was evaluated, using co-staining of HLA-ABC and C-Peptide and immunofluorescence microscopy. Compared to ctrl-ductal EVs, cyto-ductal EVs increased the mean fluorescence intensity of beta cells (**Figures 4D, E**); the effect was modest (average fold change 1.11) but consistent across ductal EV donors. Overall, these data indicate that EndoC-βH1 cells and primary human beta cells show increased HLA Class I expression after exposure to 50 µg/mL cyto-ductal EVs.

### 3.5 Fractions 1–3 from SEC do not contain TNF-α, IL-1β, and IFN-γ.

**Figure 5:**
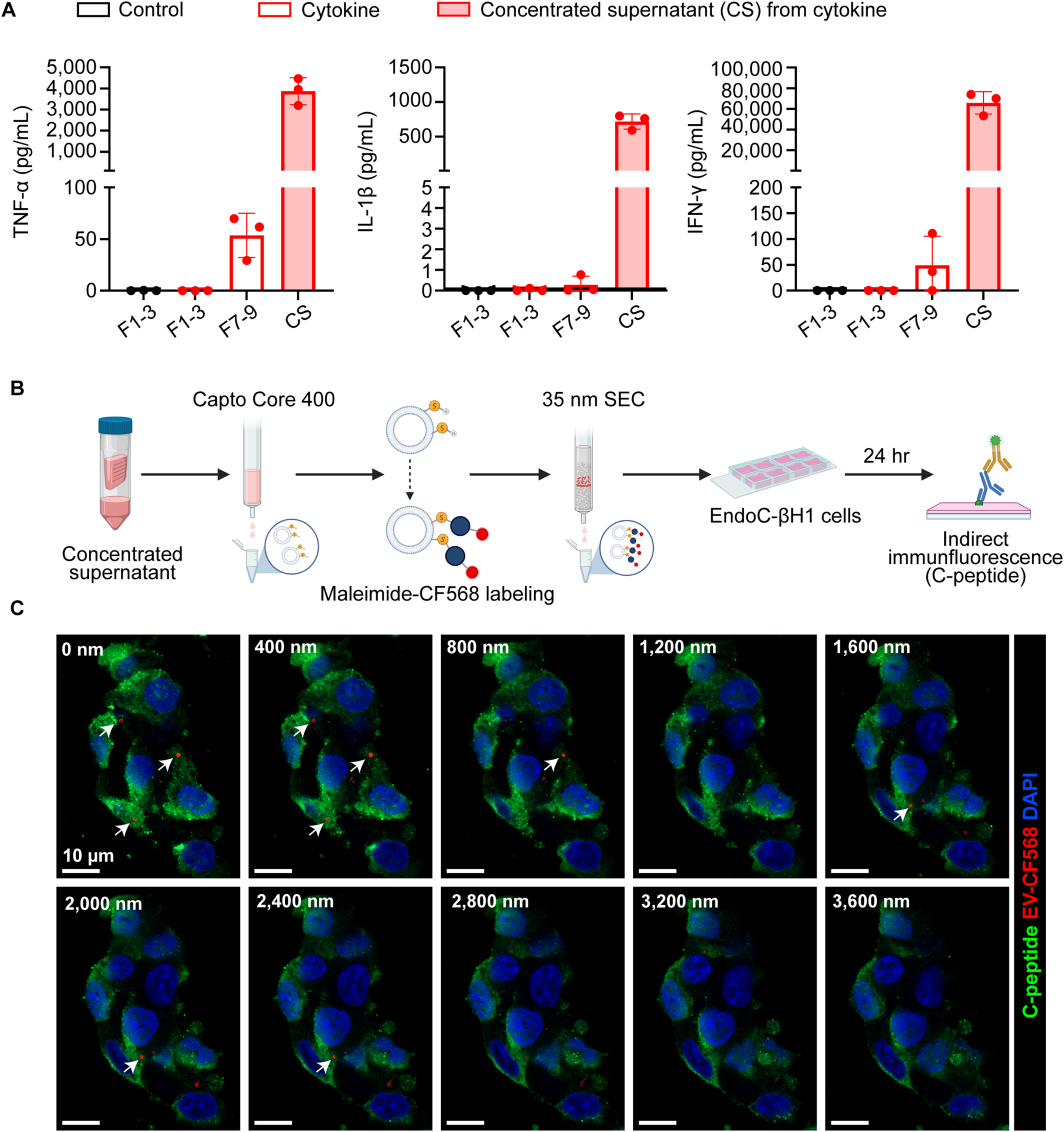
Cyto-ductal EVs are not contaminated with TNF-α, IL-1β, and IFN-γ and can be uptaken by EndoC-βH1 cells. **(A)** Quantification of TNF-α (left panel), IL-1β (middle panel), and IFN-γ (right panel) by high sensitivity ELISA in F1–3 ctrl-ductal EVs, F1–3 cyto-ductal EVs, F7–9 from cyto-ductal, and concentrated supernatant (CS) of conditioned medium from cyto-ductal cells. Data represent mean ± SEM from *N* = 3 donors. Each dot represents a donor, with the mean value derived from *n* = 2 technical replicates. **(B)** Experimental workflow for EV labeling and up-taking experiments. Cyto-ductal EVs were labeled with CF568 fluorescent dye and assessed for uptake in EndoC-βH1 cells. Created in BioRender. Erdem, N. (2026) https://BioRender.com/xptbkhm. **(C)** Representative images of EndoC-βH1 cells captured using a confocal microscopy, with CF568-labeled EVs, C-peptide, nuclei (DAPI) shown as red, green, and blue, respectively. The upper-left corner of each panel indicates the corresponding Z-plane distance (nm) from the first image. White arrows highlight representative CF568-labeled EVs colocalizing with C-peptide. Scale bar: 10 µm.

To confirm that increased HLA Class I in EndoC-βH1 cells was not due to the residual cytokines co-eluted in the EV-containing fractions, we measured the levels of TNF-α, IL-1β, and IFN-γ in various SEC fractions using ELISA. No detectable levels of TNF-α, IL-1β, or IFN-γ were found in SEC F1–3 from either ctrl-ductal or cyto-ductal cells (**Figure 5A**), demonstrating no contamination of cytokines in F1-3.

In the same experiments, we also measured TNF-α, IL-1β, and IFN-γ in the concentrated conditioned medium of cyto-ductal cells before SEC and in protein-enriched SEC F7-9 (**Figure 2B**) as positive controls; indeed, all three cytokines were detected (**Figure 5A**). Taken together, these results indicate that the effects of cyto-ductal EVs on EndoC-βH1 cells are not due to residual cytokines in SEC F1-3.

### 3.6 EndoC-βH1 cells uptake cyto-ductal EVs

The increase of HLA Class I molecules in EndoC-βH1 cells treated with cyto-ductal EVs suggested EV uptake. To confirm this, we labeled EV surface proteins using CF568, a red fluorescence dye conjugated to maleimide, which covalently binds to sulfhydryl (-SH) groups on cysteine residues (38) without affecting EV uptake (63). To enhance EV yield necessary for labeling protocol, Capto Core 400 chromatography resin (49) was used in this experiment to enrich EVs. The resulting EVs were incubated with maleimide-CF568, quenched, and the excess dye was removed using SEC (38). Labeled EVs were added to EndoC-βH1 cells for 24 hrs, followed by fixation and co-immunofluorescence staining for C-peptide, a *de novo* byproduct of insulin production, to identify EndoC-βH1 cells (**Figure 5B**).

Confocal microscopy revealed colocalization of CF568-labeled EVs with C-peptide (green fluorescence) across multiple Z planes (**Figure 5C**). As the Z planes progressed, discrete EV-CF568 signals faded within individual cells (**Figure 5C**, white arrows), demonstrating EV presence inside EndoC-βH1 cells.

Multiple controls were used to validate the specificity of EV uptake. First, the vehicle PBS was used to replace EVs during the maleimide-CF568 incubation step, which showed negative staining (**Supplementary Figure 4A**). This demonstrates that, without EVs, the dye itself did not cause background fluorescence or non-specific cellular uptake. The second control used C-peptide immunostaining of EndoC-βH1 cells that were not treated with EVs, which also showed negative staining in the red channel (**Supplementary Figure 4B**), excluding the possibility of spillover of fluorescence between channels. Finally, the secondary antibody-only control demonstrated that secondary antibody does not bind non-specifically to the sample or cause background signals (**Supplementary Figure 4C**). Taken together, these results demonstrate that cyto-ductal EVs can be uptaken by EndoC-βH1 cells within 24 hrs.

### 3.7 EV cargo analyses reveal enrichment of transcripts involved in inflammatory pathways in cyto-ductal EVs

To identify the cargo of EVs, we first performed proteomics analysis of cyto- and ctrl-ductal EVs purified from SEC F1–3 from four donors (a total of eight samples) (**Supplementary Figure 5A**), and a total of 2, 145 proteins were detected. Unsupervised clustering of the 2, 145 proteins revealed high donor-to-donor variability (**Supplementary Figure 5B**), and none of the proteins met the adjusted p-value threshold of 0.05 (**Supplementary Dataset 3**).

**Figure 6:**
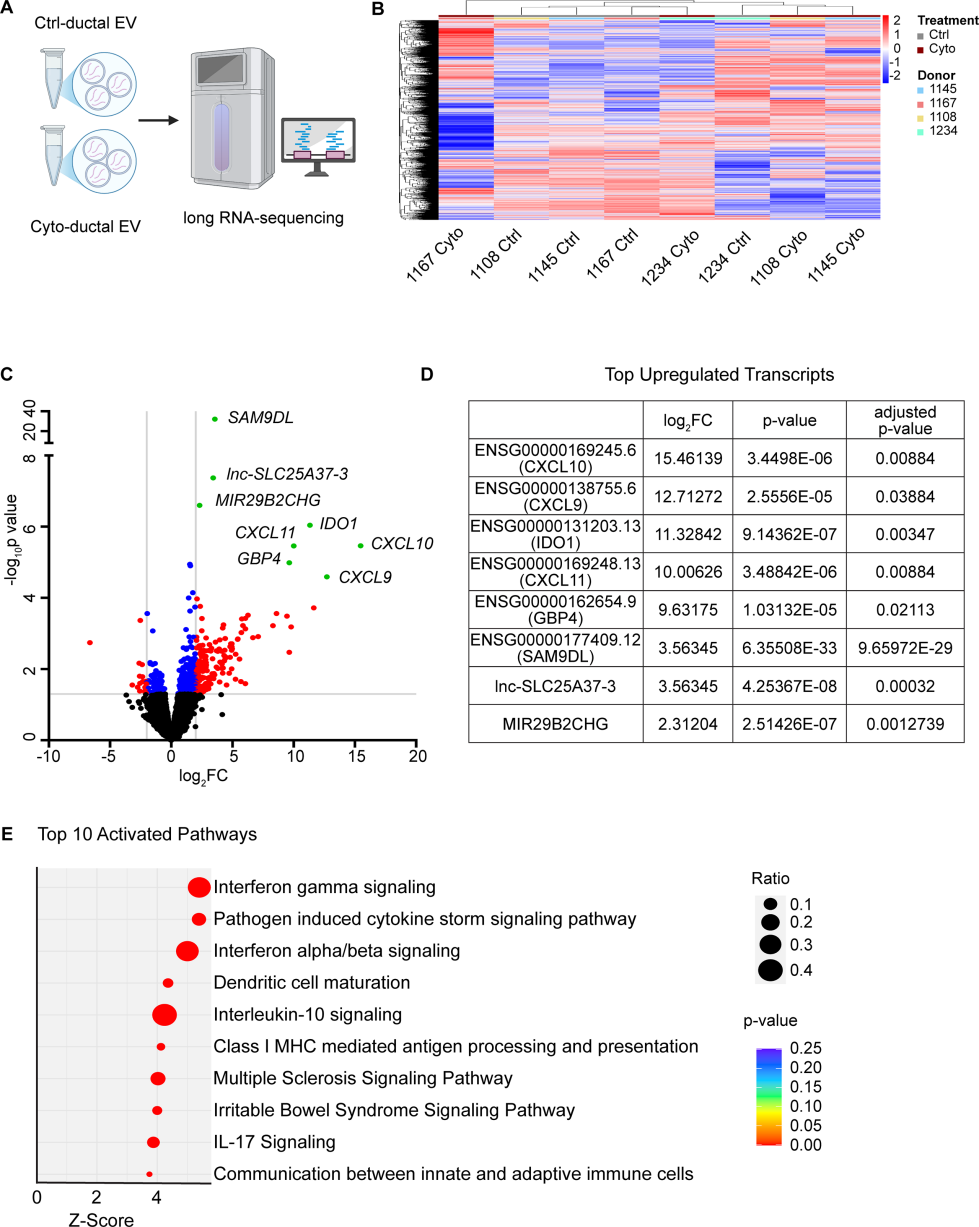
Cargo analysis of cyto-ductal EVs revealed enrichment of transcripts involved in inflammatory pathways. **(A)** Experimental workflow of long RNA-sequencing analysis of cargos in ctrl- and cyto-ductal F1–3 EVs. Created in BioRender. Erdem, N. (2026) https://BioRender.com/52anckt. *N* = 4 donors. **(B)** Unsupervised clustering of 17, 587 long RNAs found in ctrl- and cyto-ductal EVs. Z-scores are displayed as color density. *N* = 4 donors. **(C)** Volcano plot depicts long RNAs in cargos of cyto-ductal versus ctrl-ductal EVs. Each dot in the volcano plot represents a single long RNA, with the log_2_ FC plotted against the -log_10_ (p-value). Long RNAs with adjusted *p* < 0.05, *p* < 0.05, |log_2_ FC| > 2 are shown in green. Long RNAs with *p* < 0.05, |log_2_ FC| > 2 are shown in red. Long RNAs with *p* > 0.05 are represented in black. The horizontal line indicates the p-value threshold, and the vertical lines represent the log_2_ FC cutoff. *N* = 4 donors. **(D)** Top eight upregulated differentially expressed long RNAs detected in cyto- compared to ctrl-ductal EVs. *N* = 4 donors. **(E)** Ingenuity Pathway Analysis (IPA) comparing long RNAs in cyto- vs ctrl-ductal EVs. *N* = 4 donors.

Next, we performed transcriptomics analysis on the long (>200 nucleotides) and small RNAs (<200 nucleotides; typically 35–40 nucleotides) contained in the cyto- and ctrl-ductal F1–3 EVs (**Figure 6A**; **Supplementary Figure 5A**). In this experiment, the same set of four donors as the proteomics analysis were used, and a total of 17, 587 long RNA and 18, 682 small RNA sequences passed the quality control criteria and were used for subsequent analysis. Unsupervised clustering analysis revealed less donor-to-donor variability in transcriptomics analysis (**Figure 6B**; **Supplementary Figure 5C**), compared to that found for proteomics analysis (**Supplementary Figure 5B**).

In long RNA-sequencing, ENSG00000169245.6 (*CXCL10*, log_2_FC 15.461) and ENSG00000138755.6 (*CXCL9*, log_2_FC 12.713) were identified as the two most highly enriched transcripts in cyto-ductal EVs compared to ctrl-ductal EVs (**Figures 6C, D**); both genes met the criteria of *p*- and adjusted *p*-value less than 0.05. Other long RNAs that met the same statistical criteria included *IDO1*, *CXCL11*, and *GBP4* (**Figure 6D**; **Supplementary Dataset 4**). Interestingly, in small RNA sequencing, *CXCL10* (protein-coding) and *CXCL9* (protein-coding) transcripts were also found to be significantly elevated based on the *p*-value, although they did not meet the adjusted *p*-value threshold (**Supplementary Dataset 5**). We next focused on HLA Class I transcripts. In the long RNA cargo of cyto-ductal EVs, *ENSG00000206503.13 (HLA-A)*, *ENSG00000234745.14 (HLA-B)*, *ENSG00000204525.18 (HLA-C)* and *ENSG00000166710.23 (B2M)* had a log_2_FC of 2.437, 3.090, 2.654 and 1.539, respectively, compared to ctrl-ductal EVs (**Supplementary Dataset 4**). Although these transcripts reached *p*-values <0.05, none met the threshold of adjusted *p*-value <0.05.

Pathway analysis of the long RNA cargo revealed that the top activated pathways included “Interferon gamma signaling”, “Interferon alpha/beta signaling”, and “Class I MHC mediated antigen processing and presentation” (**Figure 6E**; **Supplementary Dataset 6**). Taken together, cargo analyses of cyto-ductal versus ctrl-ductal EVs demonstrate that cyto-ductal EVs are enriched for transcripts associated with inflammatory and immune-related pathways.

## 4 Discussion

Historically, the exocrine pancreas has not been examined in the context of T1D due to the minimal overlap in physiological functions between the exocrine and the endocrine compartments. However, both compartments share a common developmental origin from the foregut endoderm (64, 65), are in close physical proximity within the adult pancreas, have bi-directional blood flow (66) and are both functionally affected in pancreatic diseases, such as diabetes (15). Accordingly, in recent years, interest in studying exocrine tissue in progression of T1D has grown (67).

In T1D, the exocrine pancreas undergoes structural and functional changes (68, 69). Examples are: 1) a reduction in overall organ size (70) that may precede clinical diagnosis (71), 2) altered blood flow (72), 3) decreased enzyme production leading to digestive impairments (73, 74), and 4) increased HLA Class II expression in ductal cells of T1D donors (75), and in ductal cells treated with T1D-associated cytokines (76). These observations demonstrate an association between the exocrine pancreas and T1D progression; however, establishing causality has been challenging.

In this study, we provide evidence that the primary human ductal cells respond to T1D-associated cytokines and secrete EVs that, in turn, influence gene and protein expression in target human EndoC-βH1 beta cells and primary human islet beta cells. Specifically, we found that human ductal cells: 1) dose-dependently respond to T1D-associated proinflammatory cytokines (**Figure 1**), 2) produce EVs that can be taken up by the human EndoC-βH1 beta cells (**Figure 5**), and 3) induce upregulation of HLA Class I molecules in human EndoC-βH1 beta cells and primary human islet beta cells (**Figures 3**, **4**).

HLA Class I molecules are central to antigen presentation to CD8+ cytotoxic T lymphocytes (3–5, 77). Furthermore, increased HLA Class I expression in islet cells is a hallmark of T1D (7). Experimentally, HLA Class I expression in both human EndoC-βH1 beta cells and primary human islets can be increased by T1D-associated cytokines (78). Our current study adds EVs derived from cytokine-treated ductal cells as a novel agent capable of inducing HLA Class I expression in human EndoC-βH1 beta cells (**Figures 3**, **4**) and in primary human islet beta cells (**Figure 4**). Thus, our results suggest a role of human ductal cells in T1D progression by increasing the HLA Class I expression of beta cells via EVs.

In pancreas, beta cells and immune cells are known to secrete EVs that facilitate communication between cells (20, 79, 80). When beta cells are exposed to proinflammatory cytokines, the beta cell-derived EVs promote a proinflammatory transcriptome and dysfunction in recipient beta cells (26). These EVs also enhance the recruitment of CD8+ T cells and macrophages to the EV-treated recipient beta cells (26). EVs isolated from the EndoC-βH1 beta cells (36), following induction with an ER stress agent, also increase the expression of CD11b, HLA-DR, CD40 and CD86 on human monocytes, which suggests a role of EVs from stressed beta cells in mediating immune responses (41). Immune cells also secrete EVs that can affect beta cells; for example, EVs secreted by T cells isolated from non-obese diabetes (NOD) mice, a model that closely resembles human T1D, have been shown to increase apoptosis in isolated murine islets (81). Our findings contribute to this growing body of knowledge by demonstrating EV-mediated communication from human ductal cells to beta cells.

While previous studies focused on EVs secreted from pancreatic ductal adenocarcinoma (PDAC) or their derivative (e.g., PANC-1 cells), and the immortalized ductal cell line HPDEC (28, 82, 83), here, for the first time we isolated and characterized the EVs from primary human ductal cells. To better characterize EVs secreted from primary human ductal cells, we followed the latest recommendation from the International Society for Extracellular Vesicles (21). First, to reduce exogenous nanoparticle contamination (30), we ultracentrifuged the KnockOut Serum Replacement (KSR), a commercially-available serum-free supplement which we use for human ductal cell culture. Second, to identify EVs we characterized the eluted fractions using both EV and non-EV markers, as recommended (21). We also assessed the concentration, size, and morphology of EVs using NTA and TEM, in accordance with established guidelines (21). Third, to verify the specificity of EV action, we confirmed that SEC F1–3 did not contain residual TNF-α, IL-1β, or IFN-γ from the conditioned media of cytokine-treated ductal cells (**Figure 5A**). Taken together, these experimental optimizations ensured that the EVs derived from primary human ductal cells, whether treated with cytokines or not, are highly purified and specific, which increase confidence in the conclusion drawn from our results.

We found that cyto-ductal EVs at a concentration of 50 µg/mL induced measurable effects in recipient beta cells (**Figures 3**, **4**). This concentration falls within the range of EV doses used in other *in vitro* studies. For example, EVs produced by pancreatic islets from non-diabetic donors decreased islet amyloid polypeptide formation at a dose of 25 µg/mL (39). In another *in vitro* study, EVs from stressed EndoC-βH1 cells increased the expression of CD11b, HLA-DR, CD40, CD80 and CD86 in monocytes at a dose of 100 µg/mL (41). At present, we cannot directly assess *in vivo* relevance of the ductal EV concentrations used in our study with respect to physiological levels in the pancreatic microenvironment, because data on EV concentrations in the exocrine pancreas of non-diabetic and T1D subjects are currently not available in the literature.

A significant finding from our study is that a lower dose of proinflammatory cytokines (“250x”; 250 IU/ml TNF-α, 25 IU/ml IL-1β, and 250 IU/ml IFN-γ) is sufficient to induce the expression of a stress responsive gene *ATF3* (84) in primary human ductal cells (**Figure 1C**), and to alter the EV cargo in several long RNAs (**Figure 6**). Furthermore, in our dose-response experiments, we found that the 250x dose did not reduce ductal cell survival (**Figure 1B**). This is important because the biogenesis and function of EVs from dying cells differs from that of EVs produced by viable cells (21, 85, 86). Therefore, the 250x cytokine dose allowed us to study the effects of ductal EVs under conditions with minimum contamination of apoptotic bodies from dying cells.

The enriched cargo molecules in cyto-ductal compared to ctrl-ductal EVs included *CXCL9*, *CXCL10, CXCL11, IDO1*, *and GBP4* (**Figure 6D**). CXCL9, CXCL10, and CXCL11 are structurally related chemokine proteins that share the receptor CXCR3 (87), which is expressed by both immune cells (88, 89) and beta cells (90). Prior studies have shown that CXCL10 protein is enriched in EVs derived from the mouse MIN6 beta cell line exposed to high doses of proinflammatory cytokines; the cyto-beta EVs activate the CXCL10-CXCR3 pathway and its downstream inflammatory genes, *Cxcl10*, *Nfκb1*, and *Stat1*, leading to increased beta cell dysfunction in mouse islets (26). Furthermore, STAT1-dependent HLA Class I upregulation has been reported in head and neck cancer cells (91), and a positive correlation between STAT1 and HLA Class I expression has been observed in beta cells from individuals with T1D (7). These findings raise the possibility that the *CXCL10* transcript enriched in cyto-ductal EVs may contribute to the upregulation of HLA Class I in beta cells, but further studies are required.

Several limitations should be acknowledged. First, as discussed above, our data suggest an association between EV cargo, such as *CXCL10*, and the observed increase in HLA Class I expression in beta cells; however, a direct mechanistic link was not established. In addition to a possible transfer of EV protein or RNA cargo (85, 92), EV surface-associated proteins can interact with receptors on recipient cells and trigger downstream signaling without requiring cargo delivery (21, 26, 93). Second, the study was conducted using *in vitro* cultured human ductal cells, which allows the collection of EVs for characterization but is limited in reflecting the complexity of the *in vivo* microenvironment. Third, the lack of differences in the protein cargo of cyto- and ctrl-ductal EVs is likely due to a combination of technical and biological factors. The limited number of primary ductal cells from cadaveric donors restricts EV yield. Ductal cells were exposed to cytokines for only 48 hours; a longer exposure may yield more robust changes in EVs. Although only non-diabetic donors were studied, donor-to-donor heterogeneity is expected, because inter-individual variability is inherent in human samples. Differentiating these factors will require larger cohorts and increased EV input. Despite these limitations, to the best our knowledge this study provides the first evidence supporting the role of primary human ductal cells participating in cell-cell communication through their secreted EVs in the context of T1D-associated inflammatory cytokines.

**Figure 7:**
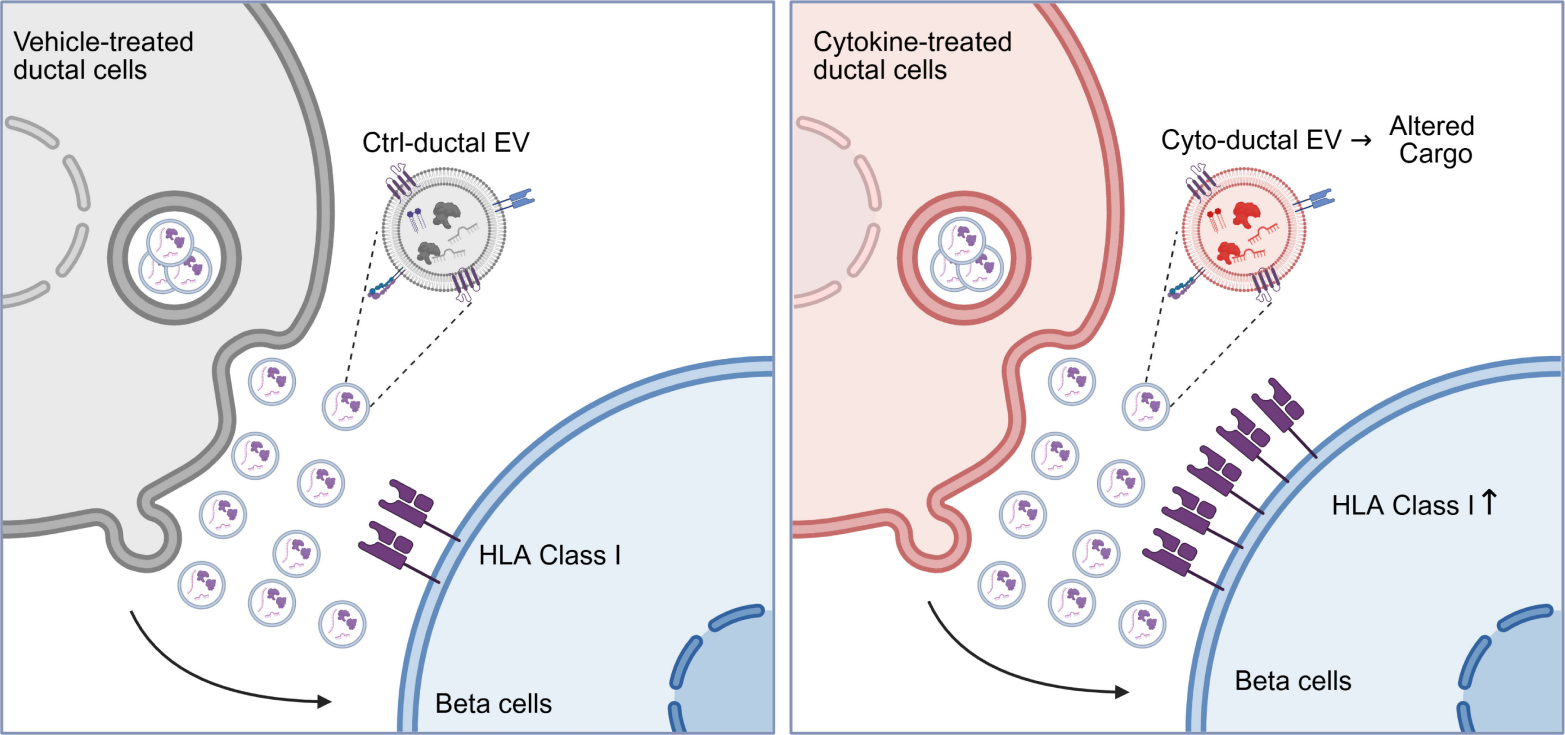
Schematic summary of key findings. Primary human ductal cells secrete EVs with altered cargo in response to T1D-associated inflammatory cytokines. These ductal EVs are up-taken by human EndoC-βH1 beta cells, leading to the upregulation of HLA Class I expression at both the transcript and protein levels. EVs from cytokine-treated ductal cells also increase the intensity of HLA Class I protein expression on primary human islet beta cells. Created in BioRender. Erdem, N. (2026) https://BioRender.com/35494bk.

In summary (**Figure 7**), our study uncovers a previously unrecognized pathway of cell-cell communication from human ductal cells mediated by EVs. Ductal cells respond to T1D-associated cytokines by secreting EVs enriched in transcripts involved in inflammatory signaling pathways. Human EndoC-βH1 beta cells and primary human islet beta cells treated with cyto-ductal EVs upregulate HLA class I molecules, which is a hallmark of T1D (7). Our results implicate ductal cells in T1D progression and inform potential targets for future therapeutic strategies.

## Data Availability

The bulk mRNA-seq of EndoC- βH1 beta cells treated with ductal EVs have been deposited to the Gene Expression Omnibus (GEO; https://www.ncbi.nlm.nih.gov/geo/) with accession number GSE316823. The long and small RNA cargo data of ductal EVs have been deposited to Synapse.org with dataset identifier with syn75903066 and syn75903067, respectively. The mass spectrometry proteomics data of ductal EVs have been deposited to the ProteomeXchange Consortium via the PRIDE partner repository with the dataset identifier PXD074059.
