# Supplementary material for: Extracellular vesicles from cytokine-treated human pancreatic ductal cells enhance HLA class I expression in beta cells
Source: Front Immunol. 2026 Jul 23;17:1788321. doi: 10.3389/fimmu.2026.1788321 (PMC13442403; doi:10.3389/fimmu.2026.1788321)
Supplement: Supplementary file 1 [file DataSheet1.pdf]

Supplementary Material

Extracellular Vesicles from Cytokine-Treated Human Pancreatic Ductal Cells Enhance HLA Class I Expression in Beta Cells

Neslihan Erdem, Heather N. Zook, Janine C. Quijano, Nathaniel P. Hansen, Joanna Palade, Eunjin Oh, Jacob Mares, Cecile Donohue, Jose A. Ortiz, Kevin Jou, Min Talley, Nagesha Guthalu Kondegowda, David Arribas-Layton, Rupangi C. Vasavada, Enrique Montero, Helena Reijonen, Debbie C. Thurmond, Patrick Pirrotte, Tijana Jovanovic-Talisman, Hsun Teresa Ku

This document contains the following information:

1. Supplementary Dataset Legends Page 2
2. Supplementary Figures and Figure legends Pages 3-8
3. Supplementary Tables Pages 9-15

1 Supplementary Dataset Legends

Suppl. Dataset 1. Genes in EndoC-βH1 cells exposed to cyto- versus ctrl-ductal EVs, related to Figure 3 and Figure S2. EndoC-βH1 cells were treated for 48 hours with EVs (50 µg/mL) derived from ductal cells treated with 250x cytokines or vehicle control, and subjected to bulk RNA sequencing analysis. N=4 donors. Genes shown met the criterion of $p < 0.05$.

Suppl. Dataset 2. Gene Set Enrichment Analysis (GSEA) using the Gene Ontology-biological process (GO-BP) database in EndoC-βH1 cells exposed to cyto- versus ctrl-ductal EVs, related to Figure 3 and Figure S2. Pathways shown met the criterion of q-value < 0.25.

Suppl. Dataset 3. Comparative analysis of protein cargo from cyto- versus ctrl-ductal EVs, related to Figure S5. Ductal cells were treated with 250x cytokines or vehicle control for 48 hrs. EVs were isolated from the conditioned media and subjected to proteomics analysis. N=4 donors.

Suppl. Dataset 4. Comparative analysis of long RNA cargo from cyto- versus ctrl-ductal EVs, related to Figure 6. Ductal cells were treated with 250x cytokines or vehicle control for 48 hrs. EVs were isolated from the conditioned media and subjected to transcriptomics analysis. N=4 donors. Genes that met the criterion of $p < 0.05$ are shown.

Suppl. Dataset 5. Comparative analysis of small RNA cargo from cyto- versus ctrl-ductal EVs, related to Figure S5. Ductal cells were treated with 250x cytokines or vehicle control for 48 hrs. EVs were isolated from the conditioned media and subjected to transcriptomics analysis. N=4 donors. Genes that met the criterion of $p < 0.05$ are shown.

Suppl. Dataset 6. Ingenuity Pathway Analysis (IPA) identifies enriched pathways in long RNA cargo from cyto- versus ctrl-ductal EVs, related to Figure 6. Long RNAs with $p < 0.05$ (Supplementary Dataset 5) were subjected to IPA analysis. Pathways that met the criterion of $p < 0.05$ are shown.

2 Supplementary Figures and Legends

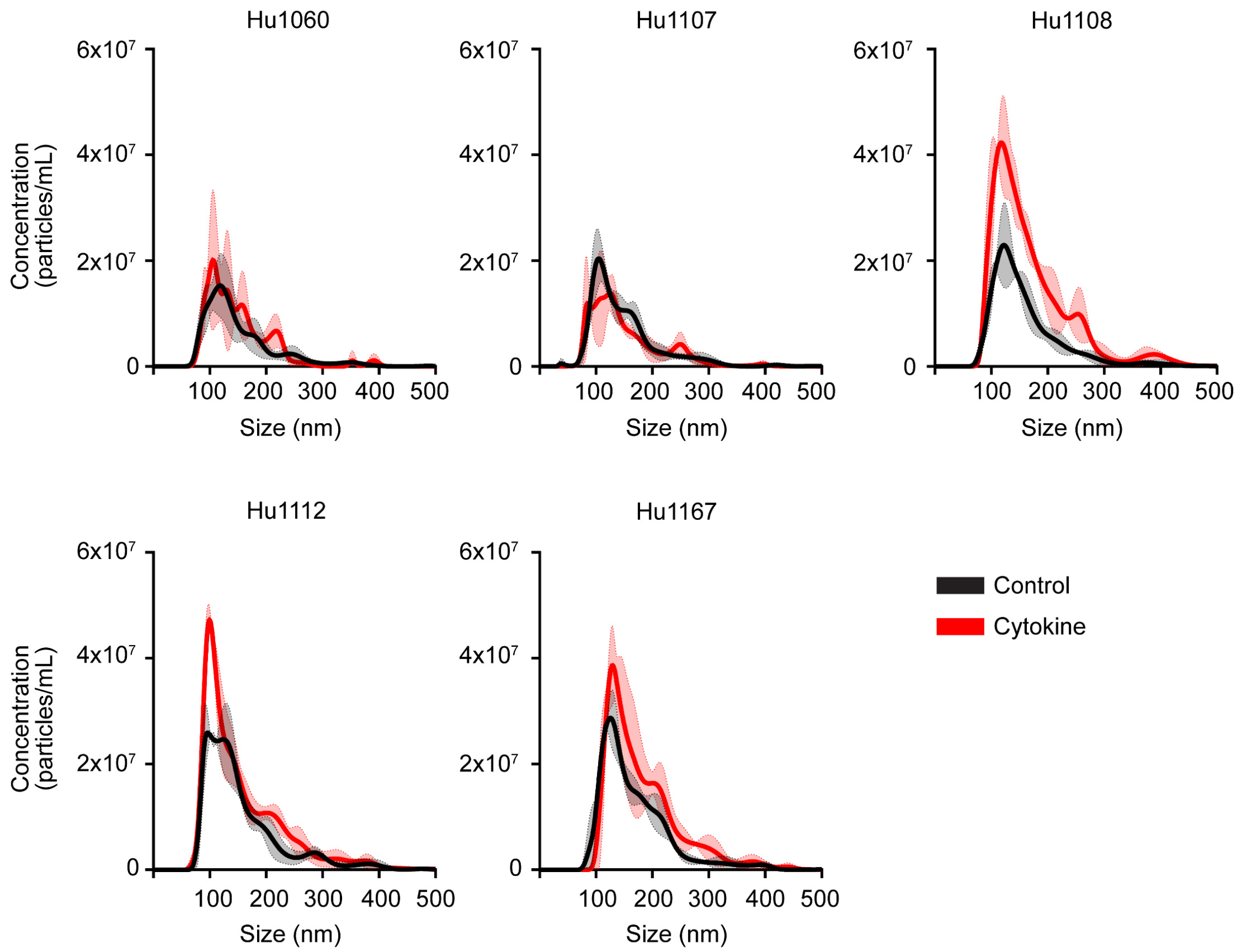

Figure S1. Nanoparticle tracking analysis, related to Fig. 2.

Size distribution by nanoparticle tracking analysis (NTA) of the combined fractions (F)1-3 from size exclusion chromatography. Each graph represents a unique donor (*N*=5). Data represent mean ± SD of *n*=3 technical replicates.

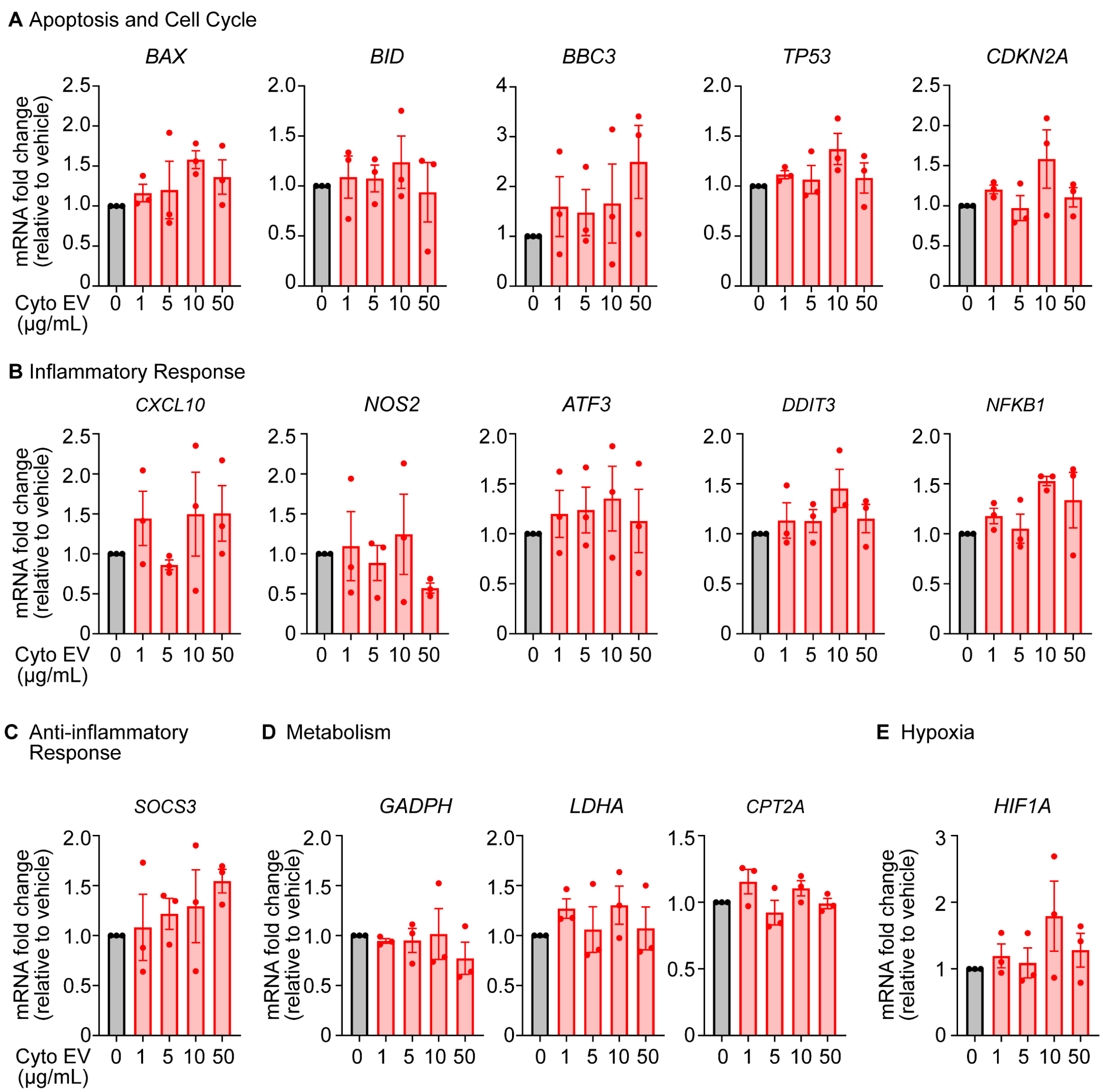

Figure S2. Expression of genes evaluated by microfluidic qRT-PCR analysis, related to Fig. 3.

Microfluidic qRT-PCR analysis was used to detect gene expression of markers in EndoC-βH1 cells receiving various doses of cyto-ductal EVs, which included: **(A)** apoptosis and cell cycle, **(B)** inflammatory response, **(C)** anti-inflammatory response, **(D)** metabolism, and **(E)** hypoxia. N=3 donors. Each dot represents a donor, with the mean derived from n=3 technical replicates. Levels of mRNA expression were normalized to housekeeping gene β-actin (*ACTB*) and are presented as fold change relative to vehicle control. Data represent mean ± SEM. One-way ANOVA with Tukey's test was used to determine significance.

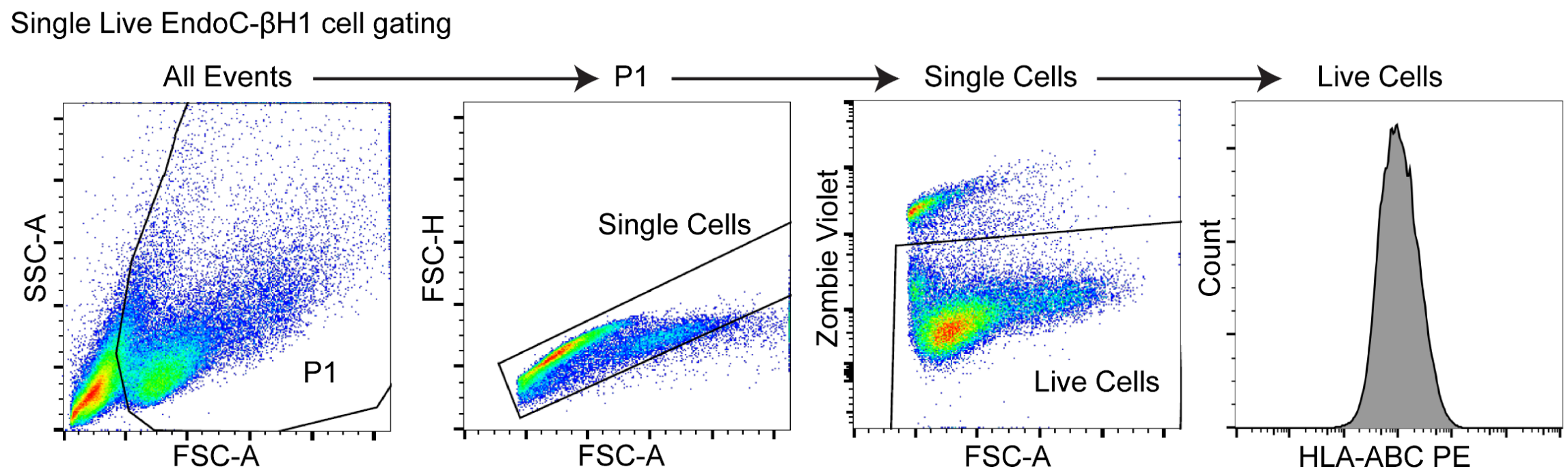

Figure S3. Gating strategy used in flow cytometry, related to Fig. 4.

Representative plots showing the sequential gating of EndoC-βH1 cells.

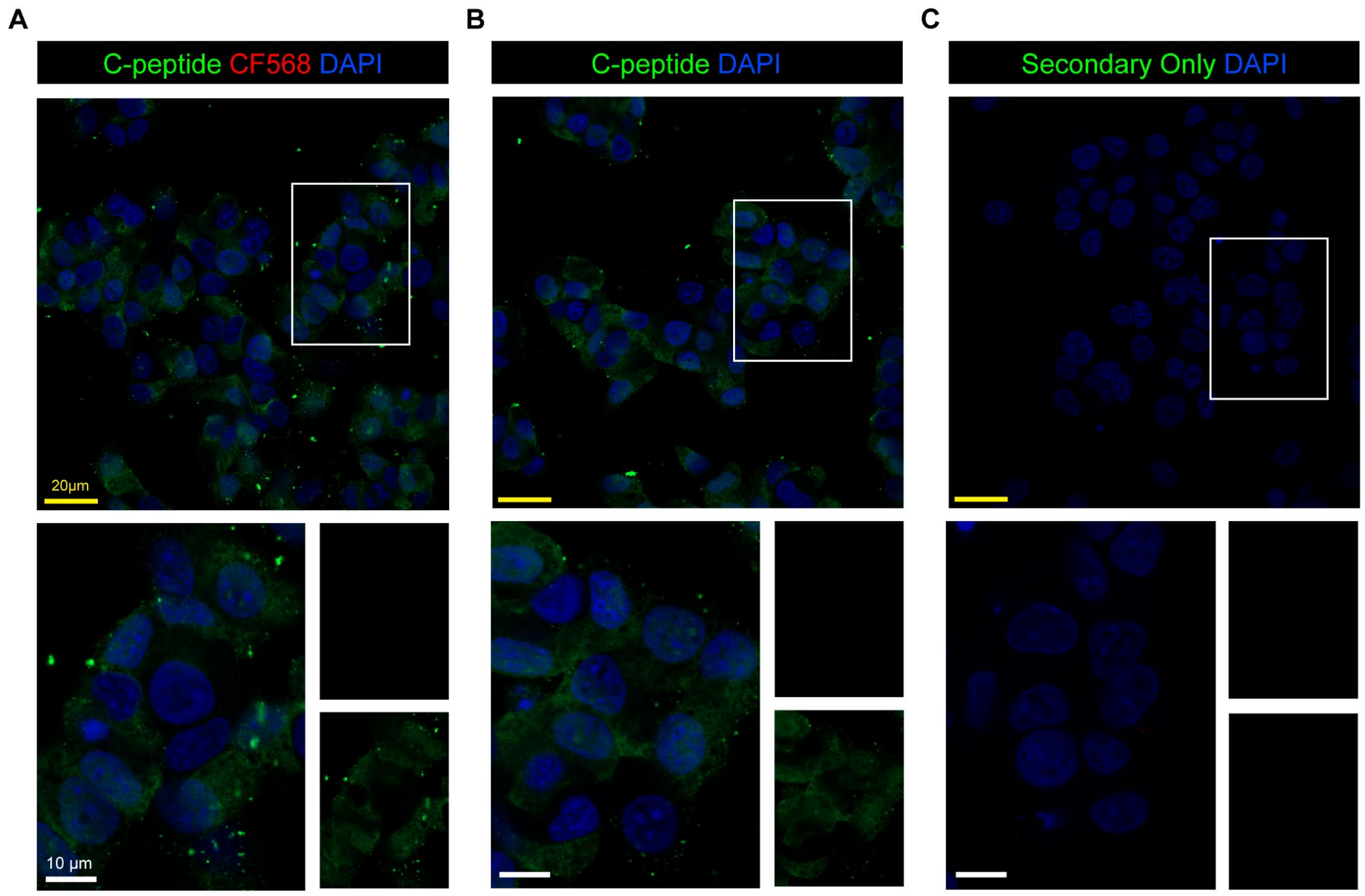

Figure S4. Negative controls for EV uptake analysis in EndoC-βH1 cells, related to Fig. 5.

(A) Vehicle (PBS) was used instead of cyto-ductal EVs at the maleimide/CF568 labeling step. Colors shown: CF568 in PBS (red), C-peptide (green), nuclei (DAPI, blue).

(B) EndoC-βH1 cells without EV-CF568 or CF568 (in PBS) treatment. Colors shown: C-peptide (green), nuclei (DAPI, blue).

(C) EndoC-βH1 cells without EV-CF568 or PBS-CF568 treatment and were stained only with secondary antibody. Colors shown: Secondary only (green), nuclei (DAPI, blue). Yellow scale bar: 20 μm. White scale bar: 10 μm.

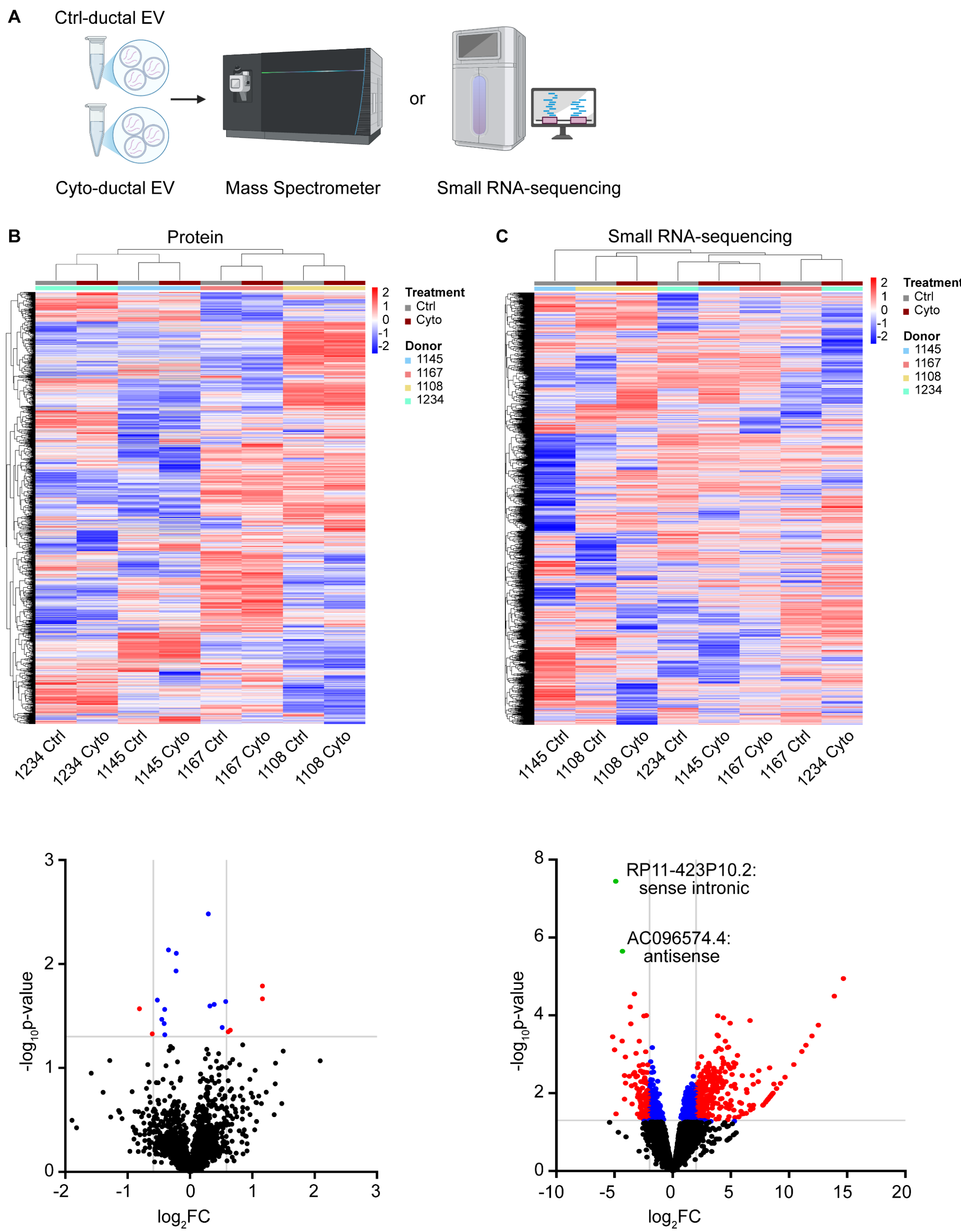

Figure S5. Analysis for proteins and small RNAs in cargo of ctrl- versus cyto-ductal EVs.

(A) Experimental workflow. EVs from ctrl- and cyto-ductal cells were subjected to proteomics or small RNA sequencing analysis. Created in BioRender. Erdem, N. (2026) https://BioRender.com/cvwv0cf.

(B) (upper panel) Unsupervised clustering of 1,695 proteins found in all eight samples. Z-scores are indicated by color density. N=4 donors. **(lower panel)** Volcano plot depicting proteins in cargos of cyto-ductal versus ctrl-ductal EVs. Each dot represents a single protein, with the $\log_2$ FC plotted against the $-\log_{10}$ (p-value). Proteins with $p < 0.05$, $|\log_2 FC| > 0.58$ are shown in red. Proteins with $p > 0.05$ are represented in black. The horizontal line indicates the p-value threshold, and the vertical lines represent the $\log_2$ FC cutoff.

(C) (upper panel) Unsupervised clustering of 18,862 small RNAs found in ctrl- and cyto-ductal EVs. Z-scores are indicated by color density. **(lower panel)** Volcano plot depicts small RNAs in cargos of cyto-ductal versus ctrl-ductal EVs. Each dot represents a single small RNA, with the $\log_2$ FC plotted against the $-\log_{10}$ (p-value). Small RNAs with adjusted $p < 0.05$, $p < 0.05$, $|\log_2 FC| > 2$ are shown in green. Small RNAs with $p < 0.05$, $|\log_2 FC| > 2$ are shown in red. Small RNAs with $p > 0.05$ are represented in black. The horizontal line indicates the p-value threshold, and the vertical lines represent the $\log_2$FC cutoff.

3 Supplementary Tables

Table S1. Summary of pancreatic exocrine tissue donor characteristics.

	**Mean ± SD (*N*=17 donors)**	**Lower limit**	**Upper limit**
Age (years)	36 ± 10	18	53
Body Mass Index (BMI) (kg/m2)	25 ± 5	20	36
HbA1c (%)	5.1 ± 0.3	4.3	5.6

Table S2. Components of 3D suspension culture medium for human ductal cells.

**Reagent**	**Final Concentration**	**Company**	**Catalog number**
DMEM/F12		Corning	10-092-CV
KSR or ED-KSR	10%	Thermo Fisher	10828-028
Nicotinamide	10 mM	Sigma-Aldrich	N0636
Gastrin II Sulfated	100 nM	Sigma-Aldrich	G1260
rm R-Spondin1	750 ng/mL	R&D Systems	3474-RS
rh EGF	50 ng/mL	R&D Systems	236-EG
rm Noggin	100 ng/mL	R&D Systems	1967-NG
A83-01	500 nM	Tocris	2939
Y-27632	10 µM	R&D Systems	1254/10

Table S3. T1D-associated proinflammatory cytokines used.

	**rh TNF-α (R&D Systems, 210-TA-005/CF)**	**rh IL-1β (R&D Systems, 201-LB-005/CF)**	**rh IFN-γ (R&D Systems, 285-IF-100/CF)**
**25x**	25 IU/mL (0.329 ng/mL)	2.5 IU/mL (0.017875 ng/mL)	25 IU/mL (1.25 ng/mL)
**100x**	100 IU/mL (1.316 ng/mL)	10 IU/mL (0.0715 ng/mL)	100 IU/mL (5 ng/mL)
**250x**	250 IU/mL (3.29 ng/mL)	25 IU/mL (0.17875 ng/mL)	250 IU/mL (12.5 ng/mL)
**500x**	500 IU/mL (6.58 ng/mL)	50 IU/mL (0.3575 ng/mL)	500 IU/mL (25 ng/mL)
**1000x**	1000 IU/mL (13.16 ng/mL)	100 IU/mL (0.715 ng/mL)	1000 IU/mL (50 ng/mL)

Table S4. List of TaqMan probes.

**Human gene**	**Assay ID**
**ACTB (β-actin)**	Hs01060665_g1
**ATF3**	Hs00231069_m1
**BAX**	Hs00180269_m1
**BAD**	Hs00188930_m1
**BBC3**	Hs00248075_m1
**BID**	Hs00609632_m1
**B2MG**	Hs00984230_m1
**CDKN2A**	Hs00923894_m1
**CPT2A**	Hs00988962_m1
**CXCL11**	Hs00171138_m1
**DDIT3**	Hs00358796_g1
**GADPH**	Hs02786624_g1
**HIF1A**	Hs00153153_m1
**HLA-A**	Hs01058806_g1
**HLA-C**	Hs00740298_g1
**LDHA**	Hs01378790_g1
**NOS2**	Hs01075529_m1

**NFKB1**	Hs00765730_m1
**SOCS3**	Hs02330328-s1
**TP53**	Hs01034249_m1

Table S5. List of antibodies.

**Antibody**	**Company, Catalog No**	**Clone**	**Host**	**RRID**	**Application (Dilution)**
Anti-CD9	Biolegend, 312105	HI9a	Mouse	AB_2075893	DB (1:1000)
Anti-CD81	Biolegend, 349502	5A6	Mouse	AB_10643417	DB (1:1000)
Anti-CD63	Novus, NBP2-42225	H5C6	Mouse	AB_2884028	DB (1:1000)
Anti-Flotillin	abcam, ab133497	EPR6041	Rabbit	AB_11156367	DB (1:500)
Anti-TSG101	Sigma-Aldrich, T5701	polyclonal	Rabbit	AB_609889	DB (1:500)
Anti-ApoA	Abcam, ab52945	EPY1368Y	Rabbit	AB_2056661	DB (1:1000)
Anti-CytC	BD Bioscience, 556432	6H2.B4	Mouse	AB_2644135	DB (1:1000)
IRDye 680RD Goat Anti-Rabbit IgG	Li-Cor, 925-68071	polyclonal	Goat	AB_2721181	DB (1:15,000)
IRDye 800CW Goat	Li-Cor, 925-32210	polyclonal	Goat	AB_2687825	DB (1:15,000)

Anti-Mouse IgG					
Anti-HLA-ABC PE	Biolegend,<br>311405	W6/32	Mouse	AB_314874	FC<br>(1:20)
Anti-C-peptide	abcam,<br>ab14181	polyclonal	Rabbit	AB_300968	IF – Figure 5, S4<br>(1:100)
Anti-HLA-ABC	Developmental Studies Hybridoma Bank, GN-ID4	GN-ID4	Rat	AB_2255626	IF – Figure 4<br>(1:100)
Alexa Fluor 488<br>Donkey<br>Anti-Rabbit IgG	Jackson Immunoresearch,<br>711-546-152	polyclonal	Donkey	AB_2340619	IF – Figure 5, S4<br>(1:500)
Alexa Fluor 488<br>Donkey<br>Anti-Rat IgG	Jackson Immunoresearch,<br>712-545-153	polyclonal	Donkey	AB_2340684	IF – Figure 4<br>(1:500)

Abbreviations: DB, dot blot; FC, flow cytometry; IF, immunofluorescence staining.
